# The PD1:PD-L1/2 Pathway from Discovery to Clinical Implementation

**DOI:** 10.3389/fimmu.2016.00550

**Published:** 2016-12-12

**Authors:** Kankana Bardhan, Theodora Anagnostou, Vassiliki A. Boussiotis

**Affiliations:** ^1^Division of Hematology-Oncology, Beth Israel Deaconess Medical Center, Harvard Medical School, Boston, MA, USA; ^2^Department of Medicine, Beth Israel Deaconess Medical Center, Harvard Medical School, Boston, MA, USA; ^3^Department of Medicine, Division of Hematology, Mayo Clinic, Rochester, MN, USA

**Keywords:** PD-1, PD-L1, T cell responses, T cell tolerance, T cell exhaustion, cancer immunology, cancer immunotherapy

## Abstract

The immune system maintains a critically organized network to defend against foreign particles, while evading self-reactivity simultaneously. T lymphocytes function as effectors and play an important regulatory role to orchestrate the immune signals. Although central tolerance mechanism results in the removal of the most of the autoreactive T cells during thymic selection, a fraction of self-reactive lymphocytes escapes to the periphery and pose a threat to cause autoimmunity. The immune system evolved various mechanisms to constrain such autoreactive T cells and maintain peripheral tolerance, including T cell anergy, deletion, and suppression by regulatory T cells (T_Regs_). These effects are regulated by a complex network of stimulatory and inhibitory receptors expressed on T cells and their ligands, which deliver cell-to-cell signals that dictate the outcome of T cell encountering with cognate antigens. Among the inhibitory immune mediators, the pathway consisting of the programed cell death 1 (PD-1) receptor (CD279) and its ligands PD-L1 (B7-H1, CD274) and PD-L2 (B7-DC, CD273) plays an important role in the induction and maintenance of peripheral tolerance and for the maintenance of the stability and the integrity of T cells. However, the PD-1:PD-L1/L2 pathway also mediates potent inhibitory signals to hinder the proliferation and function of T effector cells and have inimical effects on antiviral and antitumor immunity. Therapeutic targeting of this pathway has resulted in successful enhancement of T cell immunity against viral pathogens and tumors. Here, we will provide a brief overview on the properties of the components of the PD-1 pathway, the signaling events regulated by PD-1 engagement, and their consequences on the function of T effector cells.

## Introduction

The field of T-cell costimulation started with the “two-signal” theory of lymphocyte activation that was originally proffered to distinguish self from non-self. This model explains the process of activation or anergy when a naive T cell confronts an antigen (1, 2). As per this model, two signals from antigen-presenting cells (APCs) are required for effective activation of a naive T cell. The first signal confers specificity to the immune response and involves antigen recognition, provided by the interaction of antigenic peptide/major histocompatibility complex (MHC) with the T cell receptor (TCR). The second antigen-independent signal is the “costimulatory signal,” delivered by costimulatory molecules expressed on APCs to receptors expressed on T cells. If a T cell receives only antigen-specific TCR stimulation in the absence of costimulation, it will become unresponsive (anergic) to subsequent antigenic challenge (3, 4). Later, negative costimulatory (i.e., coinhibitory) signals were also found to exist. Receptors delivering coinhibitory signals function as immune checkpoints and play a decisive role in maintaining peripheral tolerance and impeding autoimmunity (5–8).

The best-studied pathway for T cell costimulation includes B7-1/B7-2–CD28/CTLA-4 superfamily, which is essential for T cell activation and tolerance (9–13). While both the receptors and ligands of this superfamily are structurally type I transmembrane protein with a single IgV extracellular domain that predominantly mediates the receptor–ligand interaction, the ligands also contain an IgC domain on their outer surface. The immune system functions by maintaining an intricate balance between CD28/costimulation-mediated T cell activation and CTLA-4/immune checkpoint-mediated inhibition. Identification of the programed cell death 1 (PD-1) as another inhibitory receptor and inclusion of its ligands as additional members of the B7-1/B7-2–CD28/CTLA-4 family (14, 15) re-established the importance of immune checkpoints to safeguard the maintenance of T cell tolerance. Since the beginning of its discovery, costimulation had been of therapeutic interest because it was thought to provide a way to promote T cell activation to enhance antitumor responses. But with the discovery of CTLA-4 as a potent inhibitory immune checkpoint, the notion about cancer immunotherapy was modified and the preferred approach was understood as not to activate the immune system to attack cancer but to remove the coinhibitory signals that block antitumor T cell responses. Based on the same concept, the PD-1/PD-L1 coinhibitory pathway was exploited therapeutically resulting in remarkable outcomes with 20–90% response rates in multiple clinical trials and various types of cancer (16–19).

## Discovery of the PD-1:PD-L1 Pathway

While studies have shown that PD-1–PD-L interaction is important to maintain a balance between peripheral tolerance and autoimmunity, it also impairs viral and tumor immunity, promoting chronic infection and tumor progression. PD-1 is a 288 amino acid protein mostly expressed on the surface of activated T cells (20–23). In 1992, PD-1 was identified as an apoptosis-associated molecule (24). In an attempt to identify gene(s) important for apoptosis, Tasuku Honjo and colleagues at Kyoto University performed subtractive-hybridization assay and PD-1 cDNA was found to be encoded by all of the isolated clones. However, its overexpression had no effect on apoptosis in the studied cell lines (23). In 1999, the same group demonstrated PD-1 to be a negative regulator of immune responses by studying PD-1-deficient mice, which developed an autoimmune phenotype with delayed onset, organ-specific effects and incomplete penetrance. While CTLA-4 deficiency caused the rapid-onset of systemic autoimmunity, PD-1 deficiency resulted in spontaneous development of lupus like arthritis, splenomegaly, glomerulonephritis, increased number of B-lymphocytes and myeloid cells, and increased serum IgA, IgG2b and IgG3 in C57BL/6 mice. Interestingly, PD-1 deletion in Balb/c background resulted in a distinct autoimmune phenotype as early as of 5 weeks of age, with dilated cardiomyopathy, gastritis, and high circulating level of troponin reactive IgG1. PD-1 deficiency induced subacute type I diabetes in non-obese diabetic (NOD) mice whereas lethal myocarditis was observed in mice with MRL background (8, 25–27). Introduction of the lpr mutation, which causes absence of Fas-mediated apoptosis pathway (B6-lpr/lpr-PD-1^−^/^−^), expedited the commencement and severity of the disease. However, no disease was developed in Balb/c-PD-1^−^/^−^ RAG^−^/^−^ mice, showing the importance of lymphocytes for disease development. To study the response to autoantigens, PD-1-deficient 2C TCR transgenic mice were bred to the autoreactive background (H−2^b/d^) and the offspring displayed splenomegaly, growth retardation, and lethal graft versus host disease (GVHD) (7). The group collaborated with Genetics Institute at Cambridge, MA, USA, in an attempt to identify the ligand of PD-1.

Almost in parallel, Lieping Chen’s group, then at Mayo Clinic, identified PD-L1, which was named B7-H1, as a molecule with homology to B7-1 and B7-2 (28). The group did not discover that B7-H1 is a ligand for PD-1 but reported that B7-H1 costimulates T cells *via* a receptor different from CD28, CTLA4, or ICOS and delivers an activation signal to T cells, which leads to IL-10 production, but not to detectable levels of IL-2. A third, independent research group led by Gordon Freeman at Dana–Farber Cancer Institute identified by database search a B7-like molecule that did not interact with CD28, CTLA4 or ICOS. The group collaborated with Genetics Institute at Cambridge, MA, USA, in order to identify its receptor. Through these interactions with the two independent groups, the researchers at Genetics Institute found that this B7-1 like molecule was a ligand for PD-1, and was then named PD-L1 (*Pdcd1lg1*, CD274) for PD-1 ligand 1 (14). The collaboration further identified the second PD-1 ligand, named PD-L2 (*Pdcd1lg2*, CD273) (15).

## Molecular Structure

Programed cell death 1 is composed of a single N-terminal IgV-like domain sharing 21–33% sequence identity with CTLA-4, CD28, and ICOS, about 20-amino acid stalk separating the IgV domain from the plasma membrane, a transmembrane domain, and a cytoplasmic tail containing two tyrosine-based signaling motifs. Since PD-1 lacks the membrane proximal cysteine residue, which is essential for homodimerization, it is believed to exist as monomer on the cell surface (29). Unlike CD28 and CTLA-4, PD-1 tail does not contain any SH2- or SH3-binding motifs. Instead, it contains an N-terminal sequence VDYGEL, forming an immunoreceptor tyrosine-based inhibitory motif (ITIM), which is required for recruiting SH2 domain-containing phosphatases (30) and a C-terminal sequence TEYATI, forming an immunoreceptor tyrosine-based switch motif (ITSM), essential for the inhibitory function of PD-1 (31, 32). The ligands of PD-1 (PD-L1 and PD-L2) are type I transmembrane glycoproteins, containing IgC and IgV domains. The amino acid identity between PD-L1 and PD-L2 is about 40%, while PD-Ls and B7s have about 20% similarity. Human and murine orthologs of PD-Ls display about 70% identity. The crystal structure analysis shows that PD-1 utilizes its front β-face (AGFCC’ β-strands) to bind to the β-face of PD-L1 (AGFCC’ β-face) or PD-L2 (AGFC) (29, 33, 34).

## Expression and Distribution of PD-1 and Its Ligands

Programed cell death 1 is expressed on activated CD4 and CD8 T cells, B cells, monocytes, natural killer (NK) cells, and dendritic cells (DCs) (23, 35, 36). PD-1 expression can also be induced on APCs, myeloid CD11c^+^ DCs, and monocytes (37, 38). The common γ chain cytokines interleukin-2 (IL-2), IL-7, IL-15, and IL-21 can induce PD-1 expression on T cells (21). PD-1 is expressed on CD4^−^CD8^−^ double-negative (DN) thymocytes and is essential for their selection during TCRβ rearrangement (25, 39). NFATc1 is an important transcription factor that promotes the induction of PD-1 expression following activation of T cells (40). PD-1 expression is significantly reduced by calcineurin inhibitor cyclosporine A and the NFAT-specific inhibitor VIVIT. Also mutation of an NFATc1 consensus-binding site causes complete loss of PD-1 expression in T cells. The other established transcriptional activators of PD-1 are Foxo1, Notch, and IRF9, while T-bet functions as a transcriptional repressor (20, 41–43). In macrophages, interferon (IFN)-sensitive responsive element (ISRE) and STAT1/2 regulate the constitutive and IFN-α-mediated PD-1 expression (44). PD-1 can also be selectively induced on myeloid DCs by *Listeria monocytogenes* infection or by Toll-like receptor 2 (TLR2), TLR3, TLR4, or NOD ligation, but is inhibited by IL-4 and TLR9 (45). PD-1 expression is also upregulated and sustained on “exhausted” virus-specific T cells during chronic viral infection preventing their proliferation and function in clearing the virus (46, 47).

PD-Ls have distinct expression patterns: PD-L1 is constitutively expressed on T and B cells, DCs, macrophages, mesenchymal stem cells and bone marrow-derived mast cells (35). In addition, PD-L1 is expressed on a wide variety of non-hematopoietic cells including lung, vascular endothelium, fibroblastic reticular cells, liver non-parenchymal cells, mesenchymal stem cells, pancreatic islets, astrocytes, neurons, and keratinocytes (36). It has also been shown to be expressed on placental syncytiotrophoblasts and functions in the placenta to induce fetal–maternal tolerance (48, 49). PD-L1 is expressed constitutively in the cornea and retinal pigmented epithelium (RPE) and PD-1–PD-L1 interaction protects the eye from activated T cells (50–53). In contrast, PD-L2 expression is restricted to activated DCs, macrophages, bone marrow derived mast cells, and more than 50% of peritoneal B1 cells (54). In the thymus, PD-L1 is expressed mostly in the cortex, while PD-L2 expression is confined in medullary stromal cells (55, 56). PD-L1 expression on human T cells are induced by common γ chain cytokines IL-2, IL-7, and IL-15, whereas IL-21 can stimulate PD-L1 expression on B (CD19^+^) cells from peripheral blood mononuclear cells (PBMCs). LPS or BCR activation also result in induction of PD-L1 and PD-L2 in human B cells (14, 15, 28). IFN-γ, but not tumor necrosis factor (TNF)-α, treatment results in the expression of both ligands in human monocytes. IL-10 can also induce the expression of PD-L1 on monocytes, while IL-4 and granulocyte macrophage colony-stimulating factor (GM-CSF) stimulate PD-L2 expression on DCs (57). IFN-γ can also regulate PD-L1 expression in non-lymphoid cells. Endothelial cells constitutively express PD-L1 on their surface and *in vitro* treatment with IFN-γ causes its rapid upregulation (58). In addition, MyD88, TRAF6, MEK, and JAK2 are also known to play important role in signaling pathways involved in PD-L1 expression (59–61). PD-Ls are also expressed on various tumor cells. PD-Ls mediate potent inhibitory signals after ligation with PD-1, causing a detrimental effect on antitumor immunity by allowing the tumor cells to escape immunosurveillance (62–64).

## Effects of PD-1 on Signaling Pathways

Identification of PD-Ls and confirmation of their interaction with PD-1 established PD-1 as a negative regulator of immune responses (14, 15). Unlike other members of CD28 family, PD-1 transduces signal only when cross-linked together with B- or T-cell antigen receptor. PD-1-mediated signaling inhibits T lymphocyte glucose consumption, cytokine production, proliferation, and survival. CD28 costimulation (14) or IL-2 (65) can override PD-1-mediated inhibition. PD-1 engagement prevents the expression of transcription factors associated with effector cell function, including GATA-3, T-bet, and Eomes (66). Upon TCR stimulation, the tyrosine residues in the ITIM and ITSM motifs on the cytoplasmic tail of PD-1 become phosphorylated, recruiting SHP-1 and SHP-2, which in turn, dephosphorylate proximal signaling molecules downstream of the TCR and CD28. Positional mutagenesis studies have shown that the ITSM motif is critical for the inhibitory function of PD-1 (22, 67). Specifically the ITSM tyrosine (Y248) of PD-1 associates with SHP-2 and is mandatory for PD-1-mediated inhibition of PI3K/Akt activation (22, 68). PD-1 ligation causes diminished phosphorylation of CD3, ZAP70, and protein kinase Cθ (69). It can also inhibit Erk activation, which can be overcome through IL-2, IL-7, and IL-15 signaling (70). In B cells, PD-1 engagement inhibited B cell receptor-mediated Ca^2+^ mobilization and phosphorylation of Igβ, Syk, PLC-γ2, and Erk1/2, and these effects were dependent on SHP-2 recruitment to the ITSM tyrosine of PD-1 (67).

A puzzling question has been surfaced about the role of SHP-2 versus SHP-1 in the inhibitory function of PD-1. Recruitment of SHP-2 to the cytoplasmic tail of PD-1 has been well documented in B cell line (67), Jurkat T cells (15), and primary human T cells (22, 71). SHP-1 may also be a potential candidate for interaction with PD-1 cytoplasmic tail, as found by yeast two-hybrid screening. SHP-1 functions as a negative regulator of cell activation and its expression is largely confined to hematopoietic cells (72). SHP-1-deficient mice display prolonged phosphorylation of the TCR/CD3 complex and increased activation of Lck, Fyn, and other proximal TCR signaling components (73–75). In contrast, the role of SHP-2 in T cells is different. SHP-2 is omnipresent and SHP-2 deficiency results in embryonic lethality in mice. SHP-2 mostly has been depicted as a positive regulator of cell activation and appears to be necessary for optimal induction of MAPK/Erk pathway (76). SHP-2 can recruit insulin receptor substrate to insulin receptor (77) and Grb2 to both platelet-derived growth factor receptor (78) and erythropoietin receptor (79, 80). A live cell imaging study determined that SHP-2, but not SHP-1, is the phosphatase that interacts with PD-1 upon TCR-mediated activation in live cells (81). This work showed that PD-1 is translocated to dynamic TCR microclusters and accumulates at the signaling central supramolecular activation cluster (c-SMAC). SHP-2 is shortly recruited to PD-1 thereafter in the microclusters and associates with ITSM of PD-1 (81). Using site-directed mutagenesis and stable expression of mutagenized PD-1 constructs in Jurkat T cells, it was determined that although only mutation of PD-1 Y248 abrogated interaction with SHP-2, both Y248 and Y223 are actively involved in the inhibitory effects of PD-1 on IL-2 production (82).

## Effects of PD-1 on TCR Signaling and Functional Outcomes

**Figure 1:**
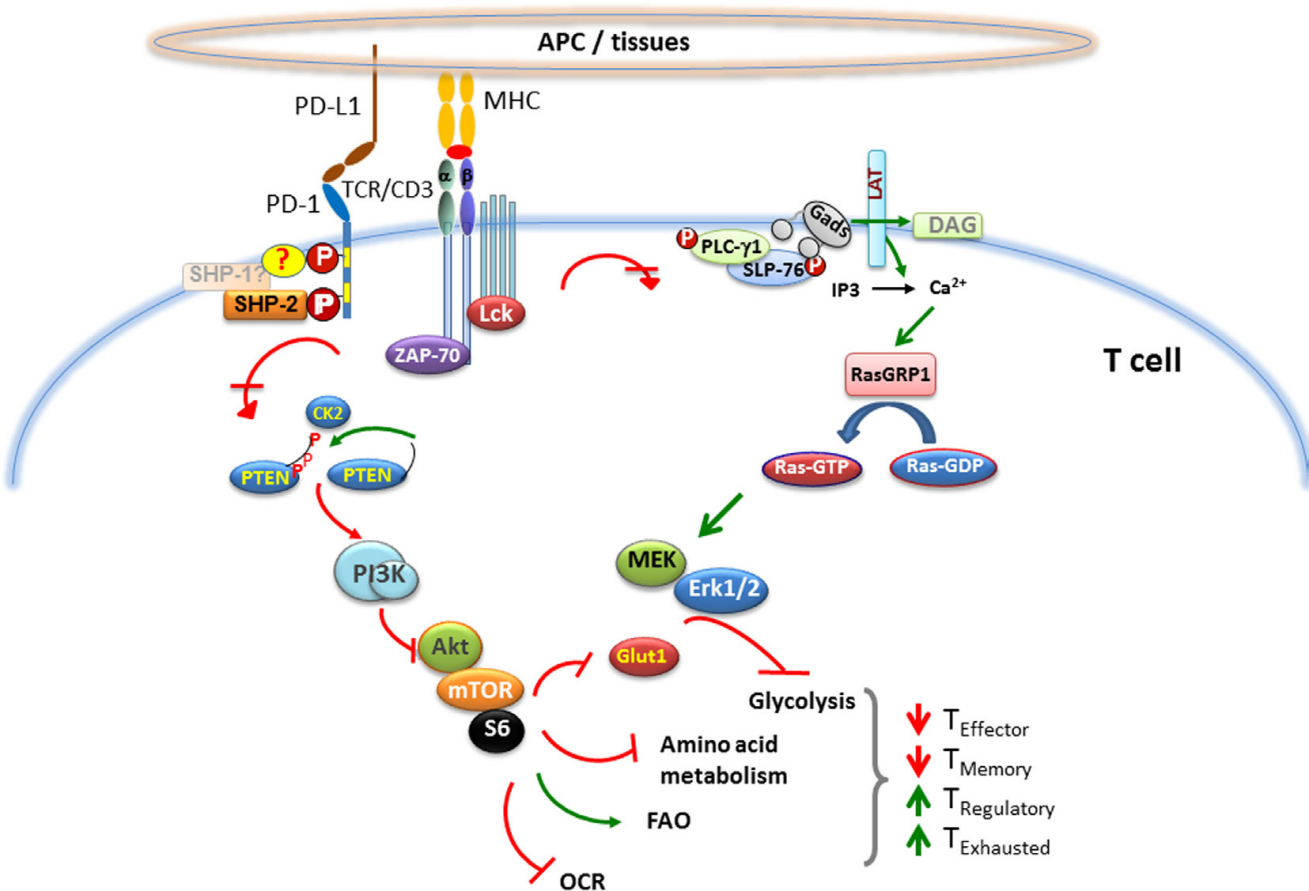
**Effect of PD-1 on major signaling pathways and subsequent metabolic reprograming in T cells**. When T cell encounters a foreign antigen presented by MHC on the surface of APC, TCR gets phosphorylated upon oligomerization of TCR/CD3 chains, followed by recruitment of activated Lck and Zap-70 to the phosphorylated ITAM (tyrosine motifs) of TCR tail, leading to initiation of downstream TCR-signaling cascade. During TCR cross-linking, when PD-1 interacts with its ligands, the two tyrosine residues on PD-1 cytoplasmic tail also become phosphorylated, and SHP-2 is recruited to ITSM (also possibly SHP-1). As a consequence, Lck and Zap-70 become dephosphorylated. PD-1 ligation also causes inhibition of PI3K/Akt/mTOR and Ras/MAPK/Erk pathways, leading to downregulation of glycolysis and amino acid metabolism and increase in fatty acid oxidation in T cells. This alteration in T cell metabolic reprograming may change the course of T cell differentiation, leading to impaired differentiation of effector and memory T cells, while enhancing the differentiation of T regulatory cells and exhausted T cells.

Programed cell death 1 ligation attenuates TCR-mediated signaling at a proximal level and impairs the activity of two signaling cascades, the PI3K/Akt and the Ras/MEK/Erk pathway (68, 83), which are co-required to initiate T cell activation (Figure 1). One of the many mechanisms *via* which PD-1 inhibits activation of the PI3K/Akt pathway includes PTEN phosphorylation and phosphatase activity, which is regulated by CK2 (84). CK2 mediates phosphorylation of PTEN C-terminus serine/threonine cluster S380/T382/T383, which, aids PTEN protein stability, while reducing PTEN lipid phosphatase activity against the substrate PIP3 (85, 86). During TCR/CD3- and CD28-mediated stimulation, PTEN is phosphorylated by CK2 (84), which stabilizes PTEN and suppresses its phosphatase activity. In contrast, PD-1 inhibits the stabilizing phosphorylation of the Ser/Thr cluster within the C-terminus domain of PTEN, resulting in increased PTEN phosphatase activity. The other major signaling pathway targeted by PD-1 is the Ras/MEK/Erk pathway (69, 83). The activation of Ras and its downstream MEK/Erk MAP kinase pathway in T cells comprises of the Ca^2+^ and DAG-mediated activation of RasGRP1 (87–89) downstream of PLC-γ1 (90), which is inhibited by PD-1 (83). Other signaling events initiated by TCR ligation are also attenuated by PD-1 ligation including activation of ZAP70 and PKCθ (69).

### PD-1 Targets the Cell Cycle

A major downstream target of the synergistic effect of PI3K/Akt and Ras/MEK/Erk activation in T cells is the cell cycle machinery. Primary T lymphocytes naturally reside in the G0 phase and lack expression of cyclins, which are required to interact with cyclin-dependent kinases (Cdks) to form cyclin–Cdk holoenzyme complexes that drive cell cycle progression (91–93). p27^kip1^, a member of the Kip/Cip family of Cdk inhibitors, interacts with Cdk2 and is abundantly present in T cells. Ubiquitin-dependent degradation of p27^kip1^ is required to initiate cell cycle progression and entry to S phase by allowing activation of Cdk2. This event is predominantly mediated by Skp1-Cullin-F-box (SCF) family ubiquitin ligase, SCF^skp2^ (94). TCR/CD3 and CD28 costimulation regulates the transcriptional induction of Skp2, a substrateSCF^skp2^ ubiquitin ligase, and this process requires simultaneous activation of PI3K/Akt and Ras/MEK/Erk pathways (95). Ligation of PD-1 during the T cell stimulation causes abrogated expression of Skp2, resulting in increased p27^kip1^ level and Cdk2 inhibition (83). The impaired Cdk2 activity inhibits Rb phosphorylation, impacting its interaction with chromatin remodeling proteins. Inhibited Cdk2 also fails to phosphorylate the checkpoint inhibitor Smad3, upregulating its transcriptional activity (96) and resulting in the increased abundance of the G1 phase Cdk inhibitor, p15^INK4B^, and the loss of the Cdk-activating phosphatase Cdc25A (83, 97).

### PD-1 Reduces the Threshold of TGF-β-Mediated Signaling

One major consequence of PD-1-mediated Cdk2 inhibition and subsequent reprograming of Smad3 transcriptional events is the conversion of naive T cells into induced T regulatory cells. Regulatory T cell populations are critical for the maintenance of peripheral tolerance, are potent inhibitors of immune responses and play an important role in the prevention of graft rejection (98, 99). Foxp3^+^ T_Regs_ can be divided into two subsets: natural T_Regs_ (nT_Regs_) and induced T_Regs_ (iT_Regs_). CD4+Foxp3+nT_Regs_ arise as committed regulatory cells from thymus (100), while iT_Regs_ (or adaptive T_Regs_) develop in the periphery from CD4^+^Foxp3^−^ naive T cells in a TGF-β- and IL-2-dependent fashion (101–107). PD-1 regulates the function of Smad3 and synergizes with TGF-β-mediated signals (83). This synergizing effect on naive T cells promotes the differentiation of T_Reg_ cells (108), thereby suppressing generation and function of T effector cells (T_EFF_) *via* a cell extrinsic mechanism. In addition, generation and function of T_Reg_ cells requires the aLb2 (LFA-1) integrin, whose activity is dependent on small GTPase Rap1 (109–112). Importantly, PD-1 does not inhibit Rap1 activation (83), indicating that PD-1 also supports the pathways required for T_Regs_ to perform their immunosuppressive functions. Experiments with PD-L1-deficient APCs resulted in minimal conversion of naive CD4^+^ T cells to iT_Regs_. PD-L1-Ig has also been shown to increase Foxp3 expression and suppressive function of established iT_Regs_ through attenuation of Akt-mTOR signaling and concomitant upregulation of PTEN signaling events that are known to drive generation of iT_Regs_ (108, 113–115).

### PD-1 Alters the Metabolic Program of Activated T Cells

Upon activation, signals from the CD28 costimulatory pathway and the γ-chain signaling cytokines promote naïve T cells to switch their metabolism from oxidative phosphorylation to glycolysis, which is required to support their growth, proliferation, and effector functions (116–119). Divergence in the metabolic reprograming is critical for imprinting distinct T cell fates. Namely, preferential switching to glycolysis accompanies effector T cell differentiation (120) and switching to fatty acid beta-oxidation (FAO) causes the conversion of T effector to T memory cells (121). Furthermore, imposing FAO by pharmacologic means boosts the generation of T_Reg_ cells (122). Studies investigating metabolism profile of T cells receiving PD-1 signals have shown that PD-1 ligation disengaged them from glycolysis, glutaminolysis, or metabolism of branched chain amino acids, but induced increased rate of FAO (123). While PD-1 ligation inhibited the expression of receptors and enzymes involved in glycolysis and glutaminolysis, it increased the expression of carnitine palmitoyl transferase (CPT1A), the rate-limiting enzyme of FAO. T cell activation causes an increase in extracellular acidification rate (ECAR), an indicator of glycolysis, and in oxygen consumption rate (OCR), an indicator of oxidative phosphorylation. PD-1 engagement results in lower ECAR and OCR, but higher OCR/ECAR ratio compared with T cells stimulated without PD-1 ligation (Figure 1). By altering the metabolic programs of T cells, PD-1 ligation seems to generate a more oxidative environment (123, 124).

## Clinical Implications of PD-1 Ligation on T Cell Immune Function

### Role of PD-1 in Chronic Viral Infection: T Cell Exhaustion

**Figure 2:**
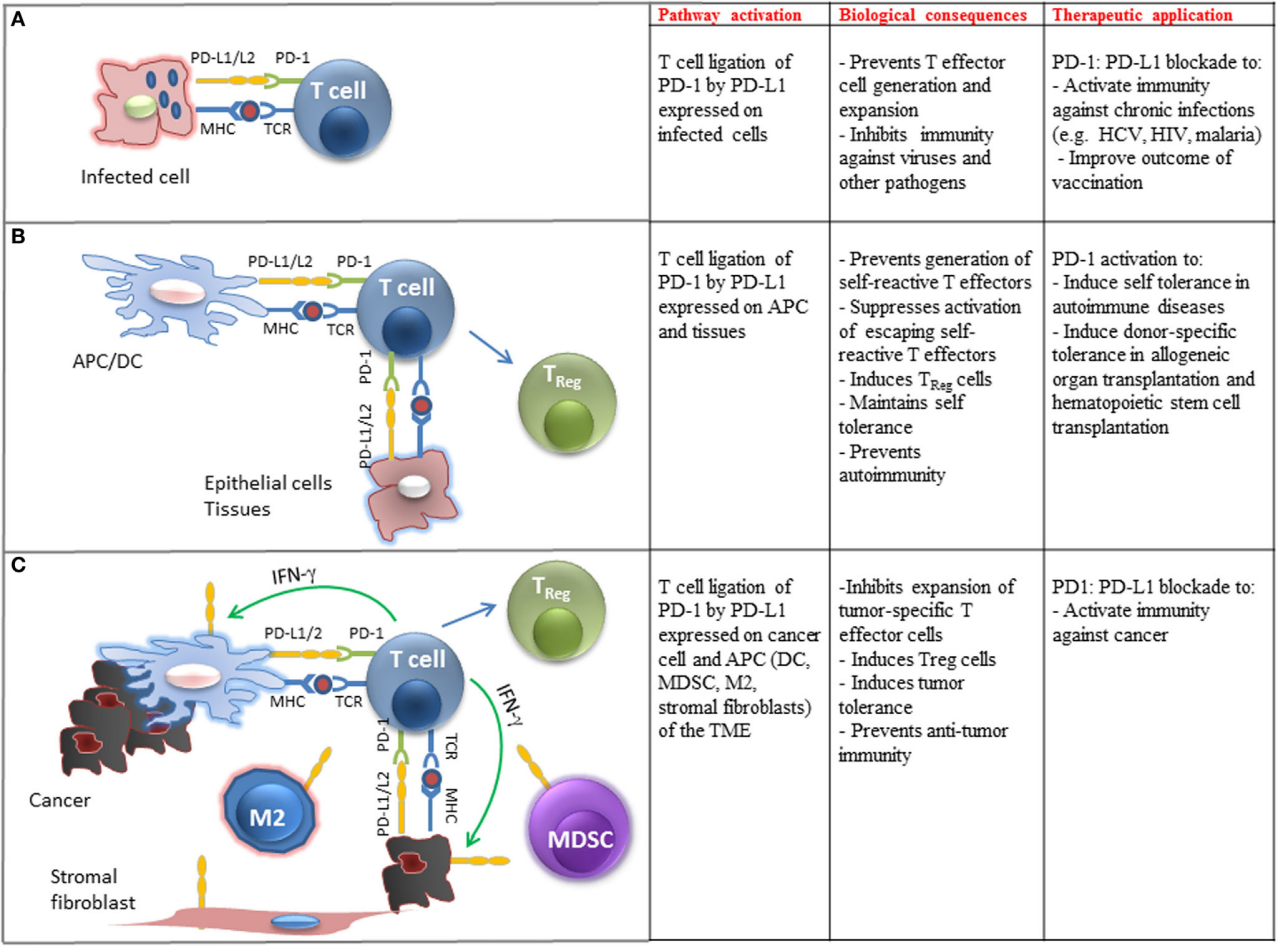
**Biological and clinical implications of PD-1 ligation on T cell immune function**. **(A)** Engagement of PD-1 by PD-L1 expressed on pathogen-presenting cells inhibits differentiation, activation, and expansion of pathogen-specific T cells in chronic infections. Therapeutic blockade of this pathway can improve pathogen-specific immunity. **(B)** Engagement of PD-1 by PD-L1 expressed on tissues and APC-presenting self-antigens prevents the generation of self-reactive T effector cells, promotes the differentiation of T_Reg_ cells, suppresses expansion of escaping self-reactive T cells, and prevents autoimmunity. Therapeutic activation of this pathway may promote transplantation tolerance and induce self-tolerance in autoimmune diseases. **(C)** Engagement of PD-1 by PD-L1 expressed on cancer cells and immune cells infiltrating the tumor microenvironment (TME) inhibits expansion of tumor-specific T cells, promotes the generation of T_Reg_ cells, promotes tumor tolerance, and suppresses antitumor immunity. Therapeutic blockade of this pathway can activate antitumor immune responses.

Programed cell death 1 has unique regulatory roles in the control of virus-specific immune responses, and these regulatory functions have been studied extensively during chronic viral infections. The CD8^+^ effector T cells behave differently in acute and chronic viral infections. During acute infection, naïve antigen-specific CD8^+^ T cells get activated, proliferate, and differentiate into effector CD8^+^ T cells and efficiently clear the virus. Most of these virus-specific effector CD8^+^ T cells then become apoptotic and a very small number (5–10%) of long-lived memory cell population arises, which is protective against secondary infection (125). However, during chronic viral infection, sustained antigenic stimulation engenders the loss of effector T cells and their failure to develop into memory CD8^+^ T cells (Figure 2A). Under these conditions, T cells become unresponsive to viral antigens and persist in a non-functional, exhausted state (T_EX_), in which they are unable to clear virus effectively (126).

During the development of exhaustion, loss of effector functions happens in a hierarchical manner: IL-2 production, high proliferative capacity and *in vitro* cytolytic activity are lost first, followed by impairment in the production of TNF-α, IFN-γ, and degranulation (127, 128). Although incapable of functioning as effector or memory, T_EX_ cells are not functionally dormant. Instead, they commit to the containment of chronic infections, because depleting CD8^+^ T cells including T_EX_ during simian immunodeficiency virus (SIV) infection results in rapid increase in viral titers and progression to AIDS (129, 130), suggesting an important role for the residual function of SIV-specific T_EX_ in maintaining a host–pathogen equilibrium and contributing to the containment of the chronic infection. T_EX_ cells often retain the capacity to produce low levels of IFN-γ and/or beta chemokines and express high levels of granzyme B. In addition, one subset of T_EX_ retains some residual cytotoxicity (127, 131, 132). High granzyme B expression is an interesting feature of T_EX_, given that the *ex vivo* killing capacity of these cells is impaired compared with T_EFF_ (131, 132). Thus, while T_EX_ cells exhibit impaired effector functions, some residual functionality remains, and this may be important in a host–pathogen equilibrium. In addition to PD-1, T_EX_ cells expressed higher levels of other inhibitory receptors (e.g., Tim-3, Lag-3, and CD160) as well (131). However, blockade of PD-1 is sufficient to induce reinvigoration of a significant fraction of this cell population, which selectively expresses T-bet^Hi^ Eomes^Lo^ PD-1^int^ and has the ability to proliferate after PD-1 blockade. In contrast, T_EX_ cells exhibiting Eomes^Hi^ PD-1^Hi^ are unable to respond after PD-1 blockade (132). Similar subsets of T_EX_ defined by reciprocal patterns of T-bet, Eomes, and/or PD-1 expression have been found in human patients with HCV and HIV infection (133, 134). In these patient populations, PD-1 blockade resulted in augmentation of pathogen-specific T cells and decrease of viral load (46, 47).

### Role of PD-1 in Transplantation and Autoimmunity

PD-L1 is expressed on a wide variety of non-hematopoietic cells and plays a key role for the maintenance of self-tolerance (Figure 2B). PD-1 and PD-L1 levels increase after heart allotransplantation and their levels correlate with the likelihood of rejection, while the use of a PD-L1-Ig fusion protein decreased rejection (135). This finding was of great interest as PD-L1 is expressed in endothelial cells, which are located between the graft cells and the immune cells and suggests a potential target to decrease the rates of graft rejection. Similarly, GvHD occurring after bone marrow transplantation (BMT) has been associated with expression of PD-1 in the infiltrating cells. However, deficiency of the PD-1 pathway has also been related to higher mortality resulting from GvHD (136, 137).

Programed cell death 1 appears to be of major clinical relevance in autoimmune diseases, such as diabetes mellitus type I (DM I) and systemic lupus erythematosus (SLE). PD-L1 is expressed in pancreatic beta islet cells and limits the activation and harmful cytotoxic function of self-reactive T-cells against islet cells, thereby protecting from autoimune damage. Treatment of non-obese mice with PD-1- and PD-L1-blocking antibodies caused faster development of DM I, while treatment with PD-L2 blocking antibody had no effect (138, 139). In SLE, data that associates polymorphisms of the PD-1 gene with susceptibility to the disease in humans (140) are in line with evidence that mice deficient in PD-1 develop manifestations that resemble SLE, including glomerulonephritis and arthritis (7). Involvement of the PD-1 pathway in other autoimmune diseases, namely multiple sclerosis, rheumatoid arthritis, and inflammatory bowel disease, is also suggested by studies in animals and attributed to either absence or non-functionality of regulatory T-cells (115).

### Role of PD-1 in Antitumor Immunity

The expression of PD-L1 and PD-L2 on APC after exposure to IFN-γ and the expression of PD-L1 in cancer cell lines (15) and primary cancer cells (141) led to the hypothesis that blockade of the PD-1:PD-L1/2 inhibitory pathway might induce antitumor immunity. The hypothesis that engagement of PD-1:PD-L1 pathway might dampen immune responses for tumors was confirmed by the observation that overexpression of PD-L1 on a mouse mastocytoma cell line inhibits CD8^+^ T cell cytolytic activity through PD-1 ligation, which intensifies tumor growth and invasiveness (142). Studies in various types of human cancers have confirmed that tumors exploit PD-1-mediated immune suppression to escape immune surveillance. A wide variety of solid tumors, including urothelial, ovarian, breast, cervical, colon, pancreatic, gastric, melanoma, glioblastoma, non-small cell lung cancer (NSCLC), and hematologic malignancies have been found to express PD-L1 and to a lesser extent PD-L2, which correlate with adverse prognosis (143–149). Importantly, the presence of PD-L1 within the tumor microenvironment (TME) also correlates with a better clinical response to PD-1/PD-L1 checkpoint blockade therapy (17, 18, 150). In addition to cancer cells, PD-L1 and PD-L2 are also expressed in other cellular components of the TME including macrophages (mostly M2), myeloid DCs, myeloid suppressor cells (MDSC), stromal fibroblasts, and endothelial cells (Figure 2C). Similarly to cancer cell-specific expression, PD-L1 expression on tumor-infiltrating immune cells correlates with clinical responses to PD-1:PD-L1 blockade therapy. Conversely, lack of PD-L1 upregulation in tumor cells or tumor-infiltrating immune cells correlates with lack of therapeutic response and disease progression (151).

PD-L1 expression on cancer cells can be mediated by cell intrinsic mechanisms activated by oncogenic mutations (152). PD-L1 expression on cancer cells and tumor-infiltrating immune cells can also be induced by local inflammation, i.e., type I/II IFN-gamma released by activated T cells, a condition termed “adaptive immune resistance” (Figure 2C) (153). It should be noted that reported studies use a different cutoff of PD-L1 expression level to define positivity and variable approaches regarding evaluation of PD-L1 expression only on cancer cells or also on tumor-infiltrating immune cells (17, 18, 150). Use of different antibodies for histopathological assessment of PD-L1 expression may also lead to variable conclusions. Regardless of these confounding factors, there is an unequivocal conclusion that the degree of PD-L1 expression in the TME positively correlates with clinical response.

**Figure 3:**
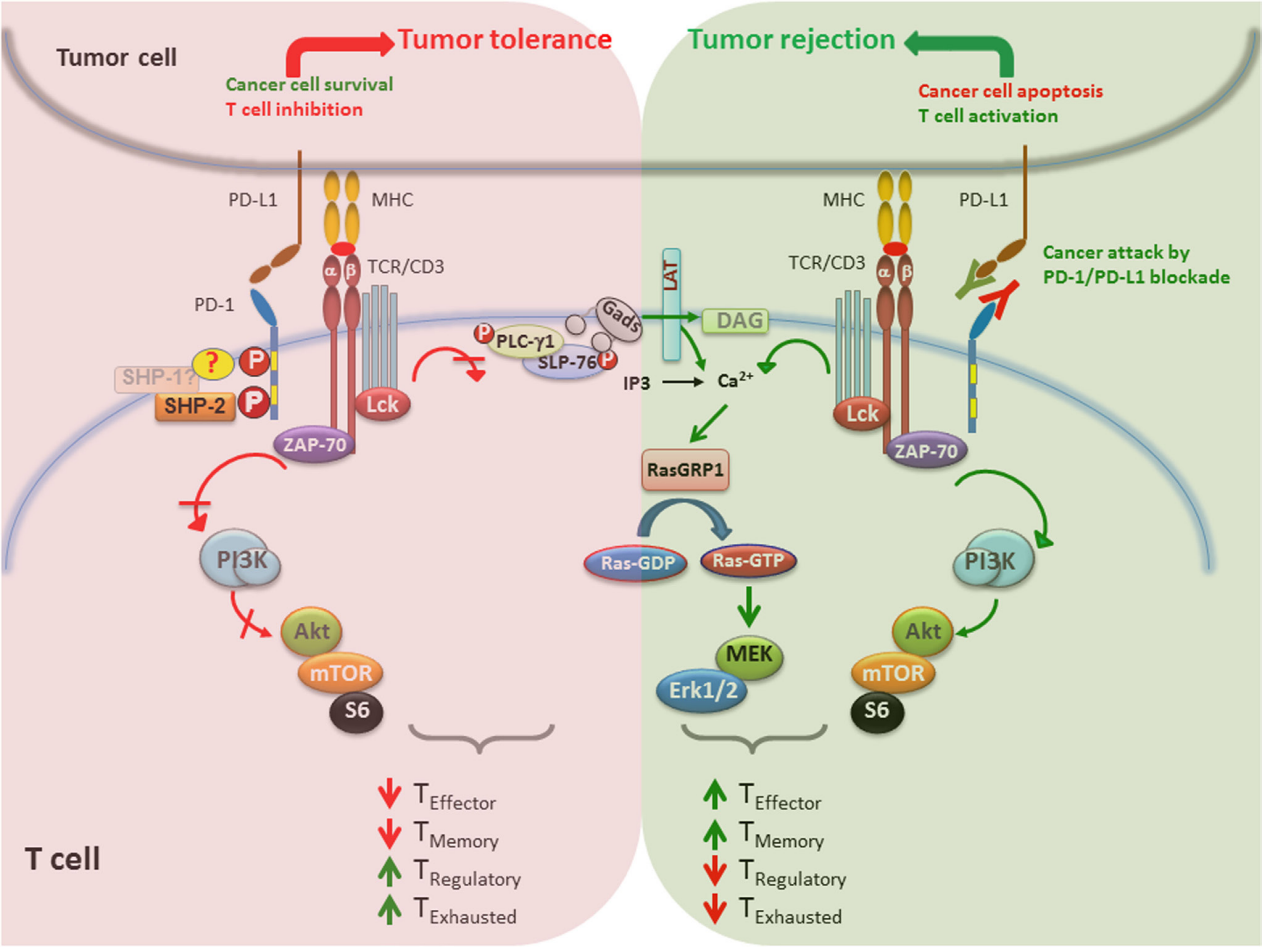
**PD-1/PD-L1 blockade enhances tumor rejection by activating T cells**. (Left) When PD-1/PD-L1 pathway is active, promotes survival of cancer cells *via* antiapoptotic signals mediated *via* PD-L1 and inhibits signaling pathways that lead to activation and expansion of T cells that recognize tumor antigens. Together, these events lead to impaired generation of T effector and memory cells and preferentiation differentiation of T_EX_ and T_Reg_ cells, which promote tumor tolerance. (Right) Blocking the PD-1/PD-L1 immune checkpoint pathway by anti-PD-1 or anti-PD-L1 antibodies suppresses cancer cell survival and enhances the antitumor responses of T cells, leading to tumor regression and rejection. In contrast to impaired TCR signaling induced by PD-1 engagement, PD-1/PD-L1 blockade causes activation of T cells by increasing PI3K/Akt or Ras/MAPK pathways, promoting differentiation of effector and memory T cells and suppression of T_EX_ and T_Reg_ differentiation.

When the PD-1/PD-L1 pathway is active in the TME, it promotes survival of cancer cells *via* antiapoptotic signals mediated *via* PD-L1 (141, 154) and inhibits the activation of signaling pathways, which are critical for survival, expansion, and differentiation of T cells that recognize tumor antigens. The imbalanced activation of signaling events in T cells results in tumor tolerance by inhibiting T effector and memory cell generation and promoting the differentiation of T_EX_ and T_Reg_ cells (Figure 3, left side). Importantly, high expression level of PD-1 has been detected on tumor-infiltrating T cells, compared with T cells in normal tissues and peripheral blood from the same patients and healthy donors, and correlate with an exhausted phenotype and an impaired effector function (155). Blocking the PD-1/PD-L1 pathway by anti-PD-1 or anti-PD-L1 antibodies suppresses cancer cell survival, reverses the effects of PD-1 on T cell signaling, and promotes the generation of T effector and memory cells while preventing the differentiation of T_EX_ and T_Reg_ cells. Together, these cell signaling and functional programs enhance antitumor T cell responses, leading to tumor regression and rejection (Figure 3, right side). It remains to be deciphered whether the therapeutic outcome of PD-1 blockade is different between patients with oncogenic PD-L1 expression versus immunogenic PD-L1 expression, in which PD-L1 is expressed on cancer cells and immune cells, respectively.

## Taking the Benchwork to Clinic

Targeted therapy against PD-1/PD-L1 has shown significant clinical activity in a variety of cancers including solid tumors and hematologic malignancies such as melanoma, renal cell carcinoma (RCC), non-small cell lung cancer (NSCLC), small cell lung cancer (SCLC), head and neck squamous cell carcinoma, gastric cancer, hepatocellular carcinoma, ovarian cancer, cervical cancer, uterine cancer, breast cancer, colorectal cancer, prostate cancer, bladder cancer, Merkel cell carcinoma, Hodgkin’s lymphoma (HL), diffuse large B cell lymphoma, and follicular lymphoma (16–19, 145, 150, 156–174).

### PD-1 Blockade in Melanoma

The use of antibodies that block immune checkpoints in the treatment of solid tumors was officially established in the armory of anticancer therapies in 2010 when ipilimumab, a CTLA-4 inhibitor, showed to improve survival in metastatic melanoma and led to the FDA approval of ipilimumab for the treatment of melanoma (175). Based on the fact that – similar to CTLA-4 – PD-1 is a coinhibitory receptor, antibodies have been developed with the goal to inhibit the PD-1:PD-L1 pathway (Table 1). These antibodies have generated remarkable responses in a wide spectrum of cancers (Table 2) and have shown better clinical benefit and better toxicity profile than CTLA4-blocking antibodies (158, 170).

**Table 1:** **Inhibitory antibodies of the PD1:PD-L1 pathway in clinical development**.

Checkpoint target	Blocking agent	Type of antibody	Developmental stage	Source
PD-1	Nivolumab (BMS-936558)	Human Ig4	FDA approved for melanoma, NSCLC, and RCC	Bristol-Myers Squibb
	Pembrolizumab (MK-3475)	Humanized IgG4	FDA approved for melanoma and NSCLC	Merck
	MEDI0680 (AMP-514)	Humanized IgG4	Phase I	Medimmune
PD-L1	Durvalumab (MEDI4736)	Human IgG4	Phase III	Medimmune
	Atezolizumab (MPDL-3280A)	Human IgG1	Phase III	Genentech
	MDX-1105/BMS-936559	Human IgG4	Phase I	Bristol-Myers Squibb
	Avelumab (MSB0010718C)	Human IgG1	Phase II	Merck Serono

**Table 2:** **Examples of clinical trials with antibodies blocking the PD-1:PD-L1 pathway**.

Cancer types	Blocking agents	Clinical response rate
Melanoma	Nivolumab	12.8% in treatment-refractory metastatic melanoma, 28% in advanced melanoma, 40% in melanoma treated in combination with ipilimumab, 20% in nivolumab followed by iplimumab, 40% in previously untreated melanoma without BRAF mutation, 57.6% (nivolumab plus iplimumab) versus 19% (ipilimumab) versus 43.7% (nivolumab) in untreated stage III or IV melanoma
	Pembrolizumab	38% in comparison to chemotherapy (14%), 26% in ipilimumab-refractory advanced melanoma, 33% in comparison to ipilimumab (11.9%) in advanced melanoma
	Atezolizumab	21% objective response rate
	MDX-1105	17.3% objective response rate

NSCLC	Nivolumab	12.8% in treatment-refractory metastatic NSCLC, 18% in advanced NSCLC, 14.5% in refractory NSCLC, 17% in previously treated NSCLC, 20% in advanced squamous cell NSCLC, higher overall survival (12.2 months) versus docetaxel treatment (6 months)
	Pembrolizumab	63 versus 0% in stage IV NSCLC patients with high and low non-synonymous mutation burden, 19.4% in advanced NSCLC of unselected population, 45.2% objective response rate in PD-L1^+^ population
	Durvalumab	14% objective response rate in unselected population and 23% in PD-L1^+^ population
	Atezolizumab	15% objective response rate in unselected population and 38% in PD-L1^+^ population
	MDX-1105	10.2% in NSCLC

Renal cell carcinoma	Nivolumab	Higher overall survival (25 months) and better objective response rate (25%) in comparison to everolimus treatment (19.6 months and 5% ORR)
	Atezolizumab	21% overall response rate
	MDX-1105	11.7% response rate

Breast cancer	Atezolizumab	19% objective response rate
	Pembrolizumab	18.5% response rate

Small cell lung cancer	Nivolumab	18% objective response rate in monotherapy and 17% objective response rate in combination
	Pembrolizumab	35% response rate
	Atezolizumab	21% objective response rate

Head and neck	Durvalumab	12% objective response rate
	Pembrolizumab	24.8% objective response rate observed in both HPV^+^ and HPV^−^ patients
	Atezolimumb	19% objective response rate

Hepatocellular carcinoma	Nivolumab	19% objective response rate

Gastric cancer	Nivolumab	31% response rate
	Atezolizumab	21% overall response rate

Ovarian cancer	Nivolumab	15% response rate, responses lasted up to 17 months
	Avelumab	14.7% objective response rate
	Pembrolizumab	11.5% response rate
	Atezolizumab	21% overall response rate
	MDX-1105	5.9% response rate

Bladder cancer	Atezolizumab	26% objective response rate in unselected population and 43% in PD-L1^+^ population
	Pembrolizumab	25% objective response rate in unselected population and 38% in PD-L1^+^ population

Mismatch repair-deficient carcinoma (colorectal and other)	Pembrolizumab	40% objective response rate in repair-deficient CRC, 0% in repair-sufficient CRC, 71% in mismatch repair-deficient non-colorectal carcinomas

Merkel cell carcinoma	Pembrolizumab	71% objective response rate

Hodgkin’s lymphoma	Nivolumab	87% objective response in relapsed or refractory Hodgkin’s lymphoma
	Pembrolizumab	66% overall response rate

Nivolumab, an IgG4 PD-1 antibody, binds to PD-1 with high affinity and specificity and was the first PD-1 blocking agent to demonstrate clinical activity in several different types of cancers, including melanoma, RCC, and NSCLC in a phase I/II trial completed in 2012 (18). In a phase Ib dose escalation study, 32% of the patients with advanced melanoma developed durable remission, which correlated with expression of PD-L1 in the tumor cells defined as positive at a minimum level of 5% (18, 168). Subsequently, in a phase III study, which compared nivolumab with dacarbazine in patients with melanoma without B-Raf mutation, nivolumab was associated with a survival benefit (73 versus 42%) and higher objective response rate (40 versus 14%). The response rate of patients with PD-L1-positive tumors (defined as PD-L1-positive tumor cells >5%) was also better than in patients with PD-L1-negative/intermediate tumors (53 versus 33%) (150). In the second phase III trial, nivolumab was compared with chemotherapy in patients with advanced melanoma non-responsive to ipilimumab (or ipililumab and BRAF inhibitor in BRAF-mutant tumors). A response rate of 32% was noted in the nivolumab treatment group versus 11% in the chemotherapy treatment group. This study also found PD-L1 as a response-predictive biomarker with 44% response rate in PD-L1 positive versus 20% in the PD-L1 negative tumors (171). Based on these outcomes, FDA approved nivolumab on December 22, 2014 for the treatment of patients with melanoma, whose disease state has progressed after prior treatment.

Pembrolizumab is a very high affinity humanized IgG4 antibody directed against human PD-1. Randomized trials have been performed in both ipilimumab naïve (NCT01866319) and previously treated patients (NCT01704287). Promising results in preliminary studies (165, 166) led to a phase II dose escalation trial comparing two dose levels of pembrolizumab to chemotherapy in ipilimumab-refractory melanoma patients (176). This study showed clear benefit for both groups that received pembrolizumab with 6-month progression-free survival (PFS) of 34 and 38% compared to the PFS of 16% of the chemotherapy group. A subsequent phase III trial compared the treatment outcome of two different administration schedules of pembrolizumab to ipilimumab in patients with advanced melanoma and provided evidence of improved survival rate in both pembrolizumab treatment groups compared to the ipilimumab group (74 and 68 versus 58%) (158). On September 4, 2014 pembrolizumab was approved by FDA for the treatment of advanced melanoma in patients previously treated with ipilimumab or a BRAF inhibitor in BRAF V600 mutation positive patients. To date, both nivolumab and pembrolizumab have also been approved by FDA and used for treatment in NSCLC, head and neck cancer, RCC, and Hodgkin lymphoma.

### PD-1 Blockade in NSCLC

The success of PD-1 blocking antibodies in NSCLC has made headlines since checkpoint blockade was thought to be responsive solely in immunogenic tumors like melanoma and RCC. A phase I dose-evaluating study of nivolumab has shown responses in patients with squamous and non-squamous histology, with or without EGFR or KRAS mutations, with or without tumor PD-L1 expression, and across different dose levels (18). On the basis of these preliminary results, two randomized phase III studies were undertaken. One compared nivolumab to docetaxel in advanced squamous NSCLC and found an improved median overall survival (OS) (9.2 versus 6.0 months) (172). In this study, tumor expression of PD-L1 determined at three different expression cutoff levels (1, 5, and 10%) had neither prognostic nor predictive of treatment benefit. The second study followed the same design and studied responses of patients with non-squamous NSCLC. In this patient group, nivolumab also resulted in improved median OS benefit compared to docetaxel (12.2 versus 9.4 months) while the OS at 1 year was 51% in the nivolumab group versus 39% in the docetaxel group (177). Correlations between therapeutic benefit and PD-L1 expression on tumor cells at the same cutoff levels (1, 5, and 10%) was also studied. In contrast to the observations in patients with advanced squamous NSCLC (172), patients with PD-L1-positive non-squamous NSCLC tumors had therapeutic benefit over those with PD-L1-negative tumors and this was observed across all three PD-L1 expression levels. Nivolumab was approved by FDA in March, 2015 for treatment of squamous NSCLC, and eventually for all patients with advanced NSCLC progressing after platinum-based chemotherapy on October 9, 2015. Almost in parallel, FDA also approved Pembrolizumab on October 2, 2015 for PD-L1-positive NSCLC, based on a large clinical trial, which assessed efficacy and safety of pembrolizumab in patients with advanced NSCLC (17). As in previous studies, therapeutic benefit was correlated with tumor PD-L1 positivity, which in this study was defined at the >50% cutoff. Importantly, this study also provided evidence that a striking survival benefit was observed in patients who received pembrolizumab without prior treatment with chemotherapy.

### PD1:PD-L1 Blockade in Other Cancers

More than 100 trials are currently investigating the use of PD-1 blockade agents as monotherapy or in combination with chemotherapeutic agents, targeted therapies, or alternate immunotherapy modalities for multiple tumor types (http://clinicaltrials.gov).

For RCC, immunotherapy has always been considered as a primary therapeutic strategy because of its immunogenic nature. The rationale for treatment with PD-1:PD-L1 blockade in RCC was further supported by the excessive PD-L1 expression in inflamed and cancerous kidney tissues (178). A randomized phase II trial comparing different doses of nivolumab in advanced RCC patients has shown a long-lasting objective response in about 22% of the patients (179). Currently, a combinatorial treatment regimen of nivolumab with either sunitinib or pazopanib is being developed, which has shown better efficacy, but higher toxicity (180). The efficacy of pembrolizumab is also currently being evaluated in a phase I/II trial in treatment naïve metastatic RCC patients in combination with pazopanib or axitinib.

One of the most impressive responses has been observed in HL, in which PD-1 blockade with nivolumab resulted in response rate of 87% (19, 181, 182). This outcome is based on the molecular upregulation of the PD-1:PD-L1 pathway through amplification of 9p24.1, which increases the gene dosage of PD-L1 and PD-L2 together with Jak2 in nodular sclerosing HL (183).

Currently, ongoing clinical trials are investigating PD-L1 blocking antibodies. Such antibodies, specifically MPDL3280A (Atezolizumab) and MEDI4736 (Durvalumab) are being evaluated in metastatic melanoma. Interestingly, Atezolizumab was associated with good responses and less pulmonary toxicity compared to PD-1 antibodies (184). In a randomized phase II trial, Atezolizumab is being compared with platinum-based chemotherapy and docetaxel after platinum failure in NSCLC. Durvalumab in combination with an EGFR inhibitor is being compared to chemoradiation in stage III NSCLC, where an objective response rate of 14% has been noticed across all histologies (185, 186). Atezolizumab is also currently being investigated as monotherapy or in combination with bevacizumab in comparison to a control group of sunitinib in treatment-naïve locally advanced or metastatic RCC. In a recent study, one complete and two partial responses were observed in patients with recurrent or metastatic triple negative breast cancer who are PD-L1 positive. PD-L1 blockade therapy also appears to be effective in bladder cancer. In a phase I study of atezolizumab in advanced bladder cancer, an objective response rate of 43% has been observed in tumors expressing high levels of PD-L1 (160).

### Combination Approaches

The combination of checkpoint blockade was first tested in advanced melanoma patients treated with nivolumab and ipilimumab and the resulting clinical activity was phenomenal (187). In a phase II study, the objective response rate for nivolumab plus ipilimumab was 59% in comparison to 11% with ipilimumab alone (188). Most recently, a phase III study of nivolumab plus ipilimumab versus nivolumab versus ipilimumab was performed in treatment naïve advanced melanoma patients (170). Again, the response rate was 57.6% for the combination therapy in comparison to 43.7% for nivolumab and 19% for ipilimumab monotherapies. The improved outcomes of the combination therapy over ipilimumab alone appear to be sustained within the 2-year follow-up of patients with combination therapy (189). The combination approach was also tested in patients with metastatic RCC. In this patient group, ipilimumab plus nivolumab in two different dose levels gave a response rate of 43 and 48%, respectively (190). Comparison between the combination checkpoint immunotherapy and sunitinib in advanced RCC is under investigation.

In NSCLC, a phase III study of nivolumab plus ipilimumab versus nivolumab monotherapy versus chemotherapy is currently undergoing (NCT02477826). Promising results were also reported from a phase I study of combination of Durvalumab and tremelimumab in NSCLC. Also, another phase III study in untreated, advanced NSCLC has recently begun with durvalumab plus tremelimumab versus durvalumab versus chemotherapy (NCT02453282). The combinatorial studies examining efficacy and safety of these drugs are also been undertaken in several other malignancies, including SCLC (191), gastric, and bladder cancer (NCT01928394). Because of different cellular expression/localization of PD-1 and PD-L1 in normal tissues, the tolerability of combination of PD-1 plus PD-L1 is also being investigated (NCT02118337).

### Determinants of Response

In order to understand how PD-1 blockade imparts tumor rejection, it is critical to identify the cell population(s) that are targeted and altered during antibody treatment (192). The presence of PD-L1 within TME in more than 1% of tumor cells has been shown to correlate with a better clinical response to PD-1/PD-L1 checkpoint blockade therapy. In contrast, lack of PD-L1 upregulation in tumor cells or lack of tumor-infiltrating immune cells has been observed in most progressing patients (18, 151, 193). However, studies in RCC have determined that detectable tumor expression of PD-L1 can be documented only in a small fraction of patients (20–30%), yet, a higher number of patients with PD-L1 negative RCC responded to PD-1 blockade (194).

As mentioned above, in addition to tumors, PD-L1 expression on tumor-infiltrating immune cells, mainly myeloid APC (macrophage and myeloid DCs) correlates with clinical responses to PD-1:PD-L1 blockade therapy (151). Based on these findings, it is possible that therapeutic PD-1 blockade might work more effectively if the tumors have already been identified by the host immune system and PD-L1 expression in cancer and innate immune cells is the consequence of local IFN-γ production by tumor-activated T cells (151, 193). Thus, one key approach to understand which cell types are important for tumor rejection is to determine location, density, and phenotype of the immune cells inside the TME and their spatiotemporal expression of PD-1 and PD-L1. Techniques to achieve this goal include, but not limited to, slide-based quantitative immunehistochemistry (IHC) and quantitative multiplexed IHC *in situ* gene expression assay (193, 195–197). The use of different anti PD-1 and anti PD-L1 antibodies, the different cutoff points to measure expression, the different cell types in which expression is being evaluated and the different scoring systems used by various pathology laboratories has caused difficulty in harmonizing the IHC readouts. The Cancer Immunotherapy Trials Network has started to review the immunodynamic effects of checkpoint inhibitors with the goal to identify and define immune assessment modalities and sites, both systemic and intratumoral, which are critical to the therapeutic success (198). Refining immune endpoints will provide the tools for the design of improved clinical trials, for selection of appropriate candidate patients for PD-1-based immunotherapy, and for assessment of induction and maintenance of therapeutic response.

## Conclusion and Future Directions

Programed cell death 1 is involved in the induction and maintenance of peripheral tolerance and plays a crucial role in the regulation of autoimmunity, transplantation immunity, infectious immunity, and tumor immunity. Currently, in parallel with the development of new discoveries about the molecular mechanisms of PD-1 function, clinical trials of combinatorial approaches are emerging. Such studies aim to maximize therapeutic antitumor benefit by blocking PD-1 together with other checkpoint inhibitors – such as CTLA-4, LAG3, TIM3, or by blocking PD-1 while engaging activating receptors of the TNF superfamily with agonist antibodies. Furthermore, PD-1 blockade together with chemoradiotherapy is anticipated to extend the therapeutic benefits of PD-1 checkpoint inhibition to a higher number of patients.

## Author Contributions

KB prepared the main body of the manuscript and figures. TA participated in the preparation of the clinical section of the manuscript. VB supervised the work and participated in the preparation of the manuscript and figures.

## Conflict of Interest Statement

The authors declare that the research was conducted in the absence of any commercial or financial relationships that could be construed as a potential conflict of interest.

## References

[1] Bretscher P Cohn M. A theory of self-nonself discrimination. Science (1970) 169:1042–9. 10.1126/science.169.3950.1042 4194660

[2] Bretscher PA. A two-step, two-signal model for the primary activation of precursor helper T cells. Proc Natl Acad Sci U S A (1999) 96(1):185–90. 10.1073/pnas.96.1.185 9874793 PMC15114

[3] Schwartz RH Mueller DL Jenkins MK Quill H T-cell clonal anergy. Cold Spring Harb Symp Quant Biol (1989) 54(Pt 2):605–10. 10.1101/SQB.1989.054.01.072 2534840

[4] Appleman LJ Boussiotis VA. T cell anergy and costimulation. Immunol Rev (2003) 192:161–80. 10.1034/j.1600-065X.2003.00009.x 12670403

[5] Tivol EA Borriello F Schweitzer AN Lynch WP Bluestone JA Sharpe AH. Loss of CTLA-4 leads to massive lymphoproliferation and fatal multiorgan tissue destruction, revealing a critical negative regulatory role of CTLA-4. Immunity (1995) 3(5):541–7. 10.1016/1074-7613(95)90125-6 7584144

[6] Waterhouse P Penninger JM Timms E Wakeham A Shahinian A Lee KP Lymphoproliferative disorders with early lethality in mice deficient in CTLA-4. Science (1995) 270:985–8. 10.1126/science.270.5238.985 7481803

[7] Nishimura H Nose M Hiai H Minato N Honjo T. Development of lupus-like autoimmune diseases by disruption of the PD-1 gene encoding an ITIM motif-carrying immunoreceptor. Immunity (1999) 11(2):141–51. 10.1016/S1074-7613(00)80089-8 10485649

[8] Nishimura H Okazaki T Tanaka Y Nakatani K Hara M Matsumori A Autoimmune dilated cardiomyopathy in PD-1 receptor-deficient mice. Science (2001) 291(5502):319–22. 10.1126/science.291.5502.319 11209085

[9] Karandikar NJ Vanderlugt CL Bluestone JA Miller SD. Targeting the B7/CD28:CTLA-4 costimulatory system in CNS autoimmune disease. J Neuroimmunol (1998) 89(1–2):10–8. 10.1016/S0165-5728(98)00058-7 9726820

[10] Oosterwegel MA Greenwald RJ Mandelbrot DA Lorsbach RB Sharpe AH. CTLA-4 and T cell activation. Curr Opin Immunol (1999) 11(3):294–300. 10.1016/S0952-7915(99)80047-8 10375557

[11] Salomon B Bluestone JA. Complexities of CD28/B7: CTLA-4 costimulatory pathways in autoimmunity and transplantation. Annu Rev Immunol (2001) 19:225–52. 10.1146/annurev.immunol.19.1.225 11244036

[12] Sansom DM CD28, CTLA-4 and their ligands: who does what and to whom? Immunology (2000) 101(2):169–77. 10.1046/j.1365-2567.2000.00121.x 11012769 PMC2327073

[13] Chambers CA Kuhns MS Egen JG Allison JP. CTLA-4-mediated inhibition in regulation of T cell responses: mechanisms and manipulation in tumor immunotherapy. Annu Rev Immunol (2001) 19:565–94. 10.1146/annurev.immunol.19.1.565 11244047

[14] Freeman GJ Long AJ Iwai Y Bourque K Chernova T Nishimura H Engagement of the PD-1 immunoinhibitory receptor by a novel B7 family member leads to negative regulation of lymphocyte activation. J Exp Med (2000) 192(7):1027–34. 10.1084/jem.192.7.1027 11015443 PMC2193311

[15] Latchman Y Wood CR Chernova T Chaudhary D Borde M Chernova I PD-L2 is a second ligand for PD-1 and inhibits T cell activation. Nat Immunol (2001) 2(3):261–8. 10.1038/85330 11224527

[16] Brahmer JR Tykodi SS Chow LQ Hwu WJ Topalian SL Hwu P Safety and activity of anti-PD-L1 antibody in patients with advanced cancer. N Engl J Med (2012) 366(26):2455–65. 10.1056/NEJMoa1200694 22658128 PMC3563263

[17] Garon EB Rizvi NA Hui R Leighl N Balmanoukian AS Eder JP Pembrolizumab for the treatment of non-small-cell lung cancer. N Engl J Med (2015) 372(21):2018–28. 10.1056/NEJMoa1501824 25891174

[18] Topalian SL Hodi FS Brahmer JR Gettinger SN Smith DC McDermott DF Safety, activity, and immune correlates of anti-PD-1 antibody in cancer. N Engl J Med (2012) 366(26):2443–54. 10.1056/NEJMoa1200690 22658127 PMC3544539

[19] Ansell SM Lesokhin AM Borrello I Halwani A Scott EC Gutierrez M PD-1 blockade with nivolumab in relapsed or refractory Hodgkin’s lymphoma. N Engl J Med (2015) 372(4):311–9. 10.1056/NEJMoa1411087 25482239 PMC4348009

[20] Terawaki S Chikuma S Shibayama S Hayashi T Yoshida T Okazaki T IFN-alpha directly promotes programmed cell death-1 transcription and limits the duration of T cell-mediated immunity. J Immunol (2011) 186(5):2772–9. 10.4049/jimmunol.1003208 21263073

[21] Kinter AL Godbout EJ McNally JP Sereti I Roby GA O’Shea MA The common gamma-chain cytokines IL-2, IL-7, IL-15, and IL-21 induce the expression of programmed death-1 and its ligands. J Immunol (2008) 181(10):6738–46. 10.4049/jimmunol.181.10.6738 18981091

[22] Chemnitz JM Parry RV Nichols KE June CH Riley JL. SHP-1 and SHP-2 associate with immunoreceptor tyrosine-based switch motif of programmed death 1 upon primary human T cell stimulation, but only receptor ligation prevents T cell activation. J Immunol (2004) 173(2):945–54. 10.4049/jimmunol.173.2.945 15240681

[23] Agata Y Kawasaki A Nishimura H Ishida Y Tsubata T Yagita H Expression of the PD-1 antigen on the surface of stimulated mouse T and B lymphocytes. Int Immunol (1996) 8(5):765–72. 10.1093/intimm/8.5.765 8671665

[24] Ishida Y Agata Y Shibahara K Honjo T. Induced expression of PD-1, a novel member of the immunoglobulin gene superfamily, upon programmed cell death. EMBO J (1992) 11(11):3887–95. 1396582 10.1002/j.1460-2075.1992.tb05481.x PMC556898

[25] Nishimura H Agata Y Kawasaki A Sato M Imamura S Minato N Developmentally regulated expression of the PD-1 protein on the surface of double-negative (CD4-CD8-) thymocytes. Int Immunol (1996) 8(5):773–80. 10.1093/intimm/8.5.773 8671666

[26] Wang J Okazaki IM Yoshida T Chikuma S Kato Y Nakaki F PD-1 deficiency results in the development of fatal myocarditis in MRL mice. Int Immunol (2010) 22(6):443–52. 10.1093/intimm/dxq026 20410257

[27] Wang J Yoshida T Nakaki F Hiai H Okazaki T Honjo T. Establishment of NOD-Pdcd1-/- mice as an efficient animal model of type I diabetes. Proc Natl Acad Sci U S A (2005) 102(33):11823–8. 10.1073/pnas.0505497102 16087865 PMC1188011

[28] Dong H Zhu G Tamada K Chen L. B7-H1, a third member of the B7 family, co-stimulates T-cell proliferation and interleukin-10 secretion. Nat Med (1999) 5(12):1365–9. 10.1038/70932 10581077

[29] Zhang X Schwartz JC Guo X Bhatia S Cao E Lorenz M Structural and functional analysis of the costimulatory receptor programmed death-1. Immunity (2004) 20(3):337–47. 10.1016/S1074-7613(04)00051-2 15030777

[30] Neel BG Gu H Pao L. The ‘Shp’ing news: SH2 domain-containing tyrosine phosphatases in cell signaling. Trends Biochem Sci (2003) 28(6):284–93. 10.1016/S0968-0004(03)00091-4 12826400

[31] Long EO. Regulation of immune responses through inhibitory receptors. Annu Rev Immunol (1999) 17:875–904. 10.1146/annurev.immunol.17.1.875 10358776

[32] Sidorenko SP Clark EA. The dual-function CD150 receptor subfamily: the viral attraction. Nat Immunol (2003) 4(1):19–24. 10.1038/ni0103-19 12496974

[33] Lázár-Molnár E Yan Q Cao E Ramagopal U Nathenson SG Almo SC. Crystal structure of the complex between programmed death-1 (PD-1) and its ligand PD-L2. Proc Natl Acad Sci U S A (2008) 105(30):10483–8. 10.1073/pnas.0804453105 18641123 PMC2492495

[34] Lin DY Tanaka Y Iwasaki M Gittis AG Su HP Mikami B The PD-1/PD-L1 complex resembles the antigen-binding Fv domains of antibodies and T cell receptors. Proc Natl Acad Sci U S A (2008) 105(8):3011–6. 10.1073/pnas.0712278105 18287011 PMC2268576

[35] Yamazaki T Akiba H Iwai H Matsuda H Aoki M Tanno Y Expression of programmed death 1 ligands by murine T cells and APC. J Immunol (2002) 169(10):5538–45. 10.4049/jimmunol.169.10.5538 12421930

[36] Keir ME Butte MJ Freeman GJ Sharpe AH. PD-1 and its ligands in tolerance and immunity. Annu Rev Immunol (2008) 26:677–704. 10.1146/annurev.immunol.26.021607.090331 18173375 PMC10637733

[37] Polanczyk MJ Hopke C Vandenbark AA Offner H. Estrogen-mediated immunomodulation involves reduced activation of effector T cells, potentiation of Treg cells, and enhanced expression of the PD-1 costimulatory pathway. J Neurosci Res (2006) 84(2):370–8. 10.1002/jnr.20881 16676326

[38] Petrovas C Casazza JP Brenchley JM Price DA Gostick E Adams WC PD-1 is a regulator of virus-specific CD8+ T cell survival in HIV infection. J Exp Med (2006) 203(10):2281–92. 10.1084/jem.20061496 16954372 PMC2118095

[39] Nishimura H Honjo T Minato N. Facilitation of beta selection and modification of positive selection in the thymus of PD-1-deficient mice. J Exp Med (2000) 191(5):891–8. 10.1084/jem.191.5.891 10704469 PMC2195853

[40] Oestreich KJ Yoon H Ahmed R Boss JM. NFATc1 regulates PD-1 expression upon T cell activation. J Immunol (2008) 181(7):4832–9. 10.4049/jimmunol.181.7.4832 18802087 PMC2645436

[41] Staron MM Gray SM Marshall HD Parish IA Chen JH Perry CJ The transcription factor FoxO1 sustains expression of the inhibitory receptor PD-1 and survival of antiviral CD8(+) T cells during chronic infection. Immunity (2014) 41(5):802–14. 10.1016/j.immuni.2014.10.013 25464856 PMC4270830

[42] Mathieu M Cotta-Grand N Daudelin JF Thébault P Labrecque N. Notch signaling regulates PD-1 expression during CD8(+) T-cell activation. Immunol Cell Biol (2013) 91(1):82–8. 10.1038/icb.2012.53 23070399

[43] Kao C Oestreich KJ Paley MA Crawford A Angelosanto JM Ali MA Transcription factor T-bet represses expression of the inhibitory receptor PD-1 and sustains virus-specific CD8+ T cell responses during chronic infection. Nat Immunol (2011) 12(7):663–71. 10.1038/ni.2046 21623380 PMC3306165

[44] Cho HY Lee SW Seo SK Choi IW Choi I Lee SW. Interferon-sensitive response element (ISRE) is mainly responsible for IFN-alpha-induced upregulation of programmed death-1 (PD-1) in macrophages. Biochim Biophys Acta (2008) 1779(12):811–9. 10.1016/j.bbagrm.2008.08.003 18771758

[45] Yao S Wang S Zhu Y Luo L Zhu G Flies S PD-1 on dendritic cells impedes innate immunity against bacterial infection. Blood (2009) 113(23):5811–8. 10.1182/blood-2009-02-203141 19339692 PMC2700320

[46] Watanabe T Bertoletti A Tanoto TA. PD-1/PD-L1 pathway and T-cell exhaustion in chronic hepatitis virus infection. J Viral Hepat (2010) 17(7):453–8. 10.1111/j.1365-2893.2010.01313.x 20487259

[47] Day CL Kaufmann DE Kiepiela P Brown JA Moodley ES Reddy S PD-1 expression on HIV-specific T cells is associated with T-cell exhaustion and disease progression. Nature (2006) 443(7109):350–4. 10.1038/nature05115 16921384

[48] Guleria I Khosroshahi A Ansari MJ Habicht A Azuma M Yagita H A critical role for the programmed death ligand 1 in fetomaternal tolerance. J Exp Med (2005) 202(2):231–7. 10.1084/jem.20050019 16027236 PMC2213002

[49] Holets LM Hunt JS Petroff MG. Trophoblast CD274 (B7-H1) is differentially expressed across gestation: influence of oxygen concentration. Biol Reprod (2006) 74(2):352–8. 10.1095/biolreprod.105.046581 16251499

[50] Hori J Wang M Miyashita M Tanemoto K Takahashi H Takemori T B7-H1-induced apoptosis as a mechanism of immune privilege of corneal allografts. J Immunol (2006) 177(9):5928–35. 10.4049/jimmunol.177.9.5928 17056517

[51] Meng Q Yang P Li B Zhou H Huang X Zhu L CD4+PD-1+ T cells acting as regulatory cells during the induction of anterior chamber-associated immune deviation. Invest Ophthalmol Vis Sci (2006) 47(10):4444–52. 10.1167/iovs.06-0201 17003438

[52] Watson MP George AJ Larkin DF. Differential effects of costimulatory pathway modulation on corneal allograft survival. Invest Ophthalmol Vis Sci (2006) 47(8):3417–22. 10.1167/iovs.05-1597 16877411

[53] Sugita S Usui Y Horie S Futagami Y Aburatani H Okazaki T T-cell suppression by programmed cell death 1 ligand 1 on retinal pigment epithelium during inflammatory conditions. Invest Ophthalmol Vis Sci (2009) 50(6):2862–70. 10.1167/iovs.08-2846 19182257

[54] Zhong X Tumang JR Gao W Bai C Rothstein TL. PD-L2 expression extends beyond dendritic cells/macrophages to B1 cells enriched for V(H)11/V(H)12 and phosphatidylcholine binding. Eur J Immunol (2007) 37(9):2405–10. 10.1002/eji.200737461 17683117

[55] Brown JA Dorfman DM Ma FR Sullivan EL Munoz O Wood CR Blockade of programmed death-1 ligands on dendritic cells enhances T cell activation and cytokine production. J Immunol (2003) 170(3):1257–66. 10.4049/jimmunol.170.3.1257 12538684

[56] Liang SC Latchman YE Buhlmann JE Tomczak MF Horwitz BH Freeman GJ Regulation of PD-1, PD-L1, and PD-L2 expression during normal and autoimmune responses. Eur J Immunol (2003) 33(10):2706–16. 10.1002/eji.200324228 14515254

[57] Selenko-Gebauer N Majdic O Szekeres A Höfler G Guthann E Korthäuer U B7-H1 (programmed death-1 ligand) on dendritic cells is involved in the induction and maintenance of T cell anergy. J Immunol (2003) 170(7):3637–44. 10.4049/jimmunol.170.7.3637 12646628

[58] Eppihimer MJ Gunn J Freeman GJ Greenfield EA Chernova T Erickson J Expression and regulation of the PD-L1 immunoinhibitory molecule on microvascular endothelial cells. Microcirculation (2002) 9(2):133–45. 10.1080/713774061 11932780 PMC3740166

[59] Lee SJ Jang BC Lee SW Yang YI Suh SI Park YM Interferon regulatory factor-1 is prerequisite to the constitutive expression and IFN-gamma-induced upregulation of B7-H1 (CD274). FEBS Lett (2006) 580(3):755–62. 10.1016/j.febslet.2005.12.093 16413538

[60] Liu J Hamrouni A Wolowiec D Coiteux V Kuliczkowski K Hetuin D Plasma cells from multiple myeloma patients express B7-H1 (PD-L1) and increase expression after stimulation with IFN-{gamma} and TLR ligands via a MyD88-, TRAF6-, and MEK-dependent pathway. Blood (2007) 110(1):296–304. 10.1182/blood-2006-10-051482 17363736

[61] Parsa AT Waldron JS Panner A Crane CA Parney IF Barry JJ Loss of tumor suppressor PTEN function increases B7-H1 expression and immunoresistance in glioma. Nat Med (2007) 13(1):84–8. 10.1038/nm1517 17159987

[62] Currie AJ Prosser A McDonnell A Cleaver AL Robinson BW Freeman GJ Dual control of antitumor CD8 T cells through the programmed death-1/programmed death-ligand 1 pathway and immunosuppressive CD4 T cells: regulation and counterregulation. J Immunol (2009) 183(12):7898–908. 10.4049/jimmunol.0901060 20007574

[63] Kuang DM Zhao Q Peng C Xu J Zhang JP Wu C Activated monocytes in peritumoral stroma of hepatocellular carcinoma foster immune privilege and disease progression through PD-L1. J Exp Med (2009) 206(6):1327–37. 10.1084/jem.20082173 19451266 PMC2715058

[64] Zhang L Gajewski TF Kline J. PD-1/PD-L1 interactions inhibit antitumor immune responses in a murine acute myeloid leukemia model. Blood (2009) 114(8):1545–52. 10.1182/blood-2009-03-206672 19417208 PMC2731636

[65] Carter L Fouser LA Jussif J Fitz L Deng B Wood CR PD-1:PD-L inhibitory pathway affects both CD4(+) and CD8(+) T cells and is overcome by IL-2. Eur J Immunol (2002) 32(3):634–43. 10.1002/1521-4141(200203)32:3<634::AID-IMMU634>3.0.CO;2-9 11857337

[66] Nurieva R Thomas S Nguyen T Martin-Orozco N Wang Y Kaja MK T-cell tolerance or function is determined by combinatorial costimulatory signals. EMBO J (2006) 25(11):2623–33. 10.1038/sj.emboj.7601146 16724117 PMC1478197

[67] Okazaki T Maeda A Nishimura H Kurosaki T Honjo T. PD-1 immunoreceptor inhibits B cell receptor-mediated signaling by recruiting src homology 2-domain-containing tyrosine phosphatase 2 to phosphotyrosine. Proc Natl Acad Sci U S A (2001) 98(24):13866–71. 10.1073/pnas.231486598 11698646 PMC61133

[68] Parry RV Chemnitz JM Frauwirth KA Lanfranco AR Braunstein I Kobayashi SV CTLA-4 and PD-1 receptors inhibit T-cell activation by distinct mechanisms. Mol Cell Biol (2005) 25(21):9543–53. 10.1128/MCB.25.21.9543-9553.2005 16227604 PMC1265804

[69] Sheppard KA Fitz LJ Lee JM Benander C George JA Wooters J PD-1 inhibits T-cell receptor induced phosphorylation of the ZAP70/CD3zeta signalosome and downstream signaling to PKCtheta. FEBS Lett (2004) 574(1–3):37–41. 10.1016/j.febslet.2004.07.083 15358536

[70] Bennett F Luxenberg D Ling V Wang IM Marquette K Lowe D Program death-1 engagement upon TCR activation has distinct effects on costimulation and cytokine-driven proliferation: attenuation of ICOS, IL-4, and IL-21, but not CD28, IL-7, and IL-15 responses. J Immunol (2003) 170(2):711–8. 10.4049/jimmunol.170.2.711 12517932

[71] Riley JL. PD-1 signaling in primary T cells. Immunol Rev (2009) 229(1):114–25. 10.1111/j.1600-065X.2009.00767.x 19426218 PMC3424066

[72] Ulyanova T Blasioli J Thomas ML. Regulation of cell signaling by the protein tyrosine phosphatases, CD45 and SHP-1. Immunol Res (1997) 16(1):101–13. 10.1007/BF02786326 9048211

[73] Plas DR Johnson R Pingel JT Matthews RJ Dalton M Roy G Direct regulation of ZAP-70 by SHP-1 in T cell antigen receptor signaling. Science (1996) 272(5265):1173–6. 10.1126/science.272.5265.1173 8638162

[74] Pani G Kozlowski M Cambier JC Mills GB Siminovitch KA. Identification of the tyrosine phosphatase PTP1C as a B cell antigen receptor-associated protein involved in the regulation of B cell signaling. J Exp Med (1995) 181(6):2077–84. 10.1084/jem.181.6.2077 7539038 PMC2192043

[75] Lorenz U Ravichandran KS Burakoff SJ Neel BG. Lack of SHPTP1 results in src-family kinase hyperactivation and thymocyte hyperresponsiveness. Proc Natl Acad Sci U S A (1996) 93(18):9624–9. 10.1073/pnas.93.18.9624 8790380 PMC38478

[76] Nguyen TV Ke Y Zhang EE Feng GS. Conditional deletion of Shp2 tyrosine phosphatase in thymocytes suppresses both pre-TCR and TCR signals. J Immunol (2006) 177(9):5990–6. 10.4049/jimmunol.177.9.5990 17056523

[77] Kharitonenkov A Schnekenburger J Chen Z Knyazev P Ali S Zwick E Adapter function of protein-tyrosine phosphatase 1D in insulin receptor/insulin receptor substrate-1 interaction. J Biol Chem (1995) 270(49):29189–93. 10.1074/jbc.270.49.29189 7493946

[78] Bennett AM Tang TL Sugimoto S Walsh CT Neel BG. Protein-tyrosine-phosphatase SHPTP2 couples platelet-derived growth factor receptor beta to Ras. Proc Natl Acad Sci U S A (1994) 91(15):7335–9. 10.1073/pnas.91.15.7335 8041791 PMC44394

[79] Maroun CR Naujokas MA Holgado-Madruga M Wong AJ Park M. The tyrosine phosphatase SHP-2 is required for sustained activation of extracellular signal-regulated kinase and epithelial morphogenesis downstream from the met receptor tyrosine kinase. Mol Cell Biol (2000) 20(22):8513–25. 10.1128/MCB.20.22.8513-8525.2000 11046147 PMC102157

[80] Tauchi T Feng GS Shen R Hoatlin M Bagby GC Jr Kabat D Involvement of SH2-containing phosphotyrosine phosphatase Syp in erythropoietin receptor signal transduction pathways. J Biol Chem (1995) 270(10):5631–5. 10.1074/jbc.270.10.5631 7534299

[81] Yokosuka T Takamatsu M Kobayashi-Imanishi W Hashimoto-Tane A Azuma M Saito T. Programmed cell death 1 forms negative costimulatory microclusters that directly inhibit T cell receptor signaling by recruiting phosphatase SHP2. J Exp Med (2012) 209(6):1201–17. 10.1084/jem.20112741 22641383 PMC3371732

[82] Chatterjee P Patsoukis N Freeman GJ Boussiotis VA Distinct roles of PD-1 ITSM and ITIM in regulating interaction of with SHP-2, ZAP-70 and Lck, and PD-1-mediated inhibitory function. Blood (2013) 122:191.

[83] Patsoukis N Brown J Petkova V Liu F Li L Boussiotis VA. Selective effects of PD-1 on Akt and Ras pathways regulate molecular components of the cell cycle and inhibit T cell proliferation. Sci Signal (2012) 5(230):ra46. 10.1126/scisignal.2002796 22740686 PMC5498435

[84] Patsoukis N Li L Sari D Petkova V Boussiotis VA. PD-1 increases PTEN phosphatase activity while decreasing PTEN protein stability by inhibiting casein kinase 2. Mol Cell Biol (2013) 33(16):3091–8. 10.1128/MCB.00319-13 23732914 PMC3753920

[85] Vazquez F Ramaswamy S Nakamura N Sellers WR. Phosphorylation of the PTEN tail regulates protein stability and function. Mol Cell Biol (2000) 20(14):5010–8. 10.1128/MCB.20.14.5010-5018.2000 10866658 PMC85951

[86] Torres J Pulido R. The tumor suppressor PTEN is phosphorylated by the protein kinase CK2 at its C terminus. Implications for PTEN stability to proteasome-mediated degradation. J Biol Chem (2001) 276(2):993–8. 10.1074/jbc.M009134200 11035045

[87] Roose JP Mollenauer M Gupta VA Stone J Weiss A. A diacylglycerol-protein kinase C-RasGRP1 pathway directs Ras activation upon antigen receptor stimulation of T cells. Mol Cell Biol (2005) 25(11):4426–41. 10.1128/MCB.25.11.4426-4441.2005 15899849 PMC1140631

[88] Ebinu JO Stang SL Teixeira C Bottorff DA Hooton J Blumberg PM RasGRP links T-cell receptor signaling to Ras. Blood (2000) 95(10):3199–203. 10807788

[89] Ebinu JO Bottorff DA Chan EY Stang SL Dunn RJ Stone JC. RasGRP, a Ras guanyl nucleotide-releasing protein with calcium- and diacylglycerol-binding motifs. Science (1998) 280(5366):1082–6. 10.1126/science.280.5366.1082 9582122

[90] Bivona TG Pérez De Castro I Ahearn IM Grana TM Chiu VK Lockyer PJ Phospholipase Cgamma activates Ras on the Golgi apparatus by means of RasGRP1. Nature (2003) 424(6949):694–8. 10.1038/nature01806 12845332

[91] Appleman LJ van Puijenbroek AA Shu KM Nadler LM Boussiotis VA. CD28 costimulation mediates down-regulation of p27kip1 and cell cycle progression by activation of the PI3K/PKB signaling pathway in primary human T cells. J Immunol (2002) 168(6):2729–36. 10.4049/jimmunol.168.6.2729 11884439

[92] Appleman LJ Berezovskaya A Grass I Boussiotis VA. CD28 costimulation mediates T cell expansion via IL-2-independent and IL-2-dependent regulation of cell cycle progression. J Immunol (2000) 164(1):144–51. 10.4049/jimmunol.164.1.144 10605005

[93] Boonen GJ van Dijk AM Verdonck LF van Lier RA Rijksen G Medema RH. CD28 induces cell cycle progression by IL-2-independent down-regulation of p27kip1 expression in human peripheral T lymphocytes. Eur J Immunol (1999) 29(3):789–98. 10.1002/(SICI)1521-4141(199903)29:03<789::AID-IMMU789>3.0.CO;2-5 10092081

[94] Carrano AC Eytan E Hershko A Pagano M. SKP2 is required for ubiquitin-mediated degradation of the CDK inhibitor p27. Nat Cell Biol (1999) 1(4):193–9. 10.1038/12013 10559916

[95] Appleman LJ Chernova I Li L Boussiotis VA. CD28 costimulation mediates transcription of SKP2 and CKS1, the substrate recognition components of SCFSkp2 ubiquitin ligase that leads p27kip1 to degradation. Cell Cycle (2006) 5(18):2123–9. 10.4161/cc.5.18.3139 16969077

[96] Matsuura I Denissova NG Wang G He D Long J Liu F. Cyclin-dependent kinases regulate the antiproliferative function of Smads. Nature (2004) 430(6996):226–31. 10.1038/nature02650 15241418

[97] Boutros R Dozier C Ducommun B. The when and wheres of CDC25 phosphatases. Curr Opin Cell Biol (2006) 18(2):185–91. 10.1016/j.ceb.2006.02.003 16488126

[98] Joffre O Santolaria T Calise D Al Saati T Hudrisier D Romagnoli P Prevention of acute and chronic allograft rejection with CD4+CD25+Foxp3+ regulatory T lymphocytes. Nat Med (2008) 14(1):88–92. 10.1038/nm1688 18066074 PMC2443705

[99] Sakaguchi S Yamaguchi T Nomura T Ono M. Regulatory T cells and immune tolerance. Cell (2008) 133(5):775–87. 10.1016/j.cell.2008.05.009 18510923

[100] Fontenot JD Rasmussen JP Williams LM Dooley JL Farr AG Rudensky AY. Regulatory T cell lineage specification by the forkhead transcription factor foxp3. Immunity (2005) 22(3):329–41. 10.1016/j.immuni.2005.01.016 15780990

[101] Fontenot JD Gavin MA Rudensky AY. Foxp3 programs the development and function of CD4+CD25+ regulatory T cells. Nat Immunol (2003) 4(4):330–6. 10.1038/ni904 12612578

[102] Hori S Nomura T Sakaguchi S. Control of regulatory T cell development by the transcription factor Foxp3. Science (2003) 299(5609):1057–61. 10.1126/science.1079490 12522256

[103] Becker C Fantini MC Schramm C Lehr HA Wirtz S Nikolaev A TGF-beta suppresses tumor progression in colon cancer by inhibition of IL-6 trans-signaling. Immunity (2004) 21(4):491–501. 10.1016/j.immuni.2004.07.020 15485627

[104] Marie JC Letterio JJ Gavin M Rudensky AY. TGF-beta1 maintains suppressor function and Foxp3 expression in CD4+CD25+ regulatory T cells. J Exp Med (2005) 201(7):1061–7. 10.1084/jem.20042276 15809351 PMC2213134

[105] Pyzik M Piccirillo CA. TGF-beta1 modulates Foxp3 expression and regulatory activity in distinct CD4+ T cell subsets. J Leukoc Biol (2007) 82(2):335–46. 10.1189/jlb.1006644 17475784

[106] Davidson TS DiPaolo RJ Andersson J Shevach EM. Cutting edge: IL-2 is essential for TGF-beta-mediated induction of Foxp3+ T regulatory cells. J Immunol (2007) 178(7):4022–6. 10.4049/jimmunol.178.7.4022 17371955

[107] Apostolou I von Boehmer H. In vivo instruction of suppressor commitment in naive T cells. J Exp Med (2004) 199(10):1401–8. 10.1084/jem.20040249 15148338 PMC2211808

[108] Francisco LM Salinas VH Brown KE Vanguri VK Freeman GJ Kuchroo VK PD-L1 regulates the development, maintenance, and function of induced regulatory T cells. J Exp Med (2009) 206(13):3015–29. 10.1084/jem.20090847 20008522 PMC2806460

[109] Wohler J Bullard D Schoeb T Barnum S. LFA-1 is critical for regulatory T cell homeostasis and function. Mol Immunol (2009) 46(11–12):2424–8. 10.1016/j.molimm.2009.04.004 19428111 PMC4627944

[110] Marski M Kandula S Turner JR Abraham C. CD18 is required for optimal development and function of CD4+CD25+ T regulatory cells. J Immunol (2005) 175(12):7889–97. 10.4049/jimmunol.175.12.7889 16339524

[111] Reedquist KA Ross E Koop EA Wolthuis RM Zwartkruis FJ van Kooyk Y The small GTPase, Rap1, mediates CD31-induced integrin adhesion. J Cell Biol (2000) 148(6):1151–8. 10.1083/jcb.148.6.1151 10725328 PMC2174316

[112] Li L Kim J Boussiotis VA. Rap1A regulates generation of T regulatory cells via LFA-1-dependent and LFA-1-independent mechanisms. Cell Immunol (2010) 266(1):7–13. 10.1016/j.cellimm.2010.08.014 20864093 PMC2966523

[113] Haxhinasto S Mathis D Benoist C. The AKT-mTOR axis regulates de novo differentiation of CD4+Foxp3+ cells. J Exp Med (2008) 205(3):565–74. 10.1084/jem.20071477 18283119 PMC2275380

[114] Strauss L Whiteside TL Knights A Bergmann C Knuth A Zippelius A. Selective survival of naturally occurring human CD4+CD25+Foxp3+ regulatory T cells cultured with rapamycin. J Immunol (2007) 178(1):320–9. 10.4049/jimmunol.178.1.320 17182569

[115] Francisco L Sage PT Sharpe AH The PD-1 pathway in tolerance and autoimmunity. Immunol Rev (2010) 236(1):219–42. 10.1111/j.1600-065X.2010.00923.x 20636820 PMC2919275

[116] Frauwirth KA Thompson CB. Regulation of T lymphocyte metabolism. J Immunol (2004) 172(8):4661–5. 10.4049/jimmunol.172.8.4661 15067038

[117] Rathmell JC Vander Heiden MG Harris MH Frauwirth KA Thompson CB. In the absence of extrinsic signals, nutrient utilization by lymphocytes is insufficient to maintain either cell size or viability. Mol Cell (2000) 6(3):683–92. 10.1016/S1097-2765(00)00066-6 11030347

[118] Frauwirth KA Riley JL Harris MH Parry RV Rathmell JC Plas DR The CD28 signaling pathway regulates glucose metabolism. Immunity (2002) 16(6):769–77. 10.1016/S1074-7613(02)00323-0 12121659

[119] Wieman HL Wofford JA Rathmell JC. Cytokine stimulation promotes glucose uptake via phosphatidylinositol-3 kinase/Akt regulation of Glut1 activity and trafficking. Mol Biol Cell (2007) 18(4):1437–46. 10.1091/mbc.E06-07-0593 17301289 PMC1838986

[120] Chang CH Curtis JD Maggi LB Jr Faubert B Villarino AV O’Sullivan D Posttranscriptional control of T cell effector function by aerobic glycolysis. Cell (2013) 153(6):1239–51. 10.1016/j.cell.2013.05.016 23746840 PMC3804311

[121] Pearce EL Walsh MC Cejas PJ Harms GM Shen H Wang LS Enhancing CD8 T-cell memory by modulating fatty acid metabolism. Nature (2009) 460(7251):103–7. 10.1038/nature08097 19494812 PMC2803086

[122] Michalek RD Gerriets VA Jacobs SR Macintyre AN MacIver NJ Mason EF Cutting edge: distinct glycolytic and lipid oxidative metabolic programs are essential for effector and regulatory CD4+ T cell subsets. J Immunol (2011) 186(6):3299–303. 10.4049/jimmunol.1003613 21317389 PMC3198034

[123] Patsoukis N Bardhan K Chatterjee P Sari D Liu B Bell LN PD-1 alters T-cell metabolic reprogramming by inhibiting glycolysis and promoting lipolysis and fatty acid oxidation. Nat Commun (2015) 6:6692. 10.1038/ncomms7692 25809635 PMC4389235

[124] Tkachev V Goodell S Opipari AW Hao LY Franchi L Glick GD Programmed death-1 controls T cell survival by regulating oxidative metabolism. J Immunol (2015) 194(12):5789–800. 10.4049/jimmunol.1402180 25972478 PMC4562423

[125] Wherry EJ Barber DL Kaech SM Blattman JN Ahmed R. Antigen-independent memory CD8 T cells do not develop during chronic viral infection. Proc Natl Acad Sci U S A (2004) 101(45):16004–9. 10.1073/pnas.0407192101 15505208 PMC524220

[126] Shin H Wherry EJ. CD8 T cell dysfunction during chronic viral infection. Curr Opin Immunol (2007) 19(4):408–15. 10.1016/j.coi.2007.06.004 17656078

[127] Wherry EJ. T cell exhaustion. Nat Immunol (2011) 12(6):492–9. 10.1038/ni.2035 21739672

[128] Wherry EJ Blattman JN Murali-Krishna K van der Most R Ahmed R. Viral persistence alters CD8 T-cell immunodominance and tissue distribution and results in distinct stages of functional impairment. J Virol (2003) 77(8):4911–27. 10.1128/JVI.77.8.4911-4927.2003 12663797 PMC152117

[129] Jin X Bauer DE Tuttleton SE Lewin S Gettie A Blanchard J Dramatic rise in plasma viremia after CD8(+) T cell depletion in simian immunodeficiency virus-infected macaques. J Exp Med (1999) 189(6):991–8. 10.1084/jem.189.6.991 10075982 PMC2193038

[130] Schmitz JE Kuroda MJ Santra S Sasseville VG Simon MA Lifton MA Control of viremia in simian immunodeficiency virus infection by CD8+ lymphocytes. Science (1999) 283(5403):857–60. 10.1126/science.283.5403.857 9933172

[131] Blackburn SD Shin H Haining WN Zou T Workman CJ Polley A Coregulation of CD8+ T cell exhaustion by multiple inhibitory receptors during chronic viral infection. Nat Immunol (2009) 10(1):29–37. 10.1038/ni.1679 19043418 PMC2605166

[132] Paley MA Kroy DC Odorizzi PM Johnnidis JB Dolfi DV Barnett BE Progenitor and terminal subsets of CD8+ T cells cooperate to contain chronic viral infection. Science (2012) 338(6111):1220–5. 10.1126/science.1229620 23197535 PMC3653769

[133] Buggert M Tauriainen J Yamamoto T Frederiksen J Ivarsson MA Michaëlsson J T-bet and Eomes are differentially linked to the exhausted phenotype of CD8+ T cells in HIV infection. PLoS Pathog (2014) 10(7):e1004251. 10.1371/journal.ppat.1004251 25032686 PMC4102564

[134] Urbani S Amadei B Tola D Massari M Schivazappa S Missale G PD-1 expression in acute hepatitis C virus (HCV) infection is associated with HCV-specific CD8 exhaustion. J Virol (2006) 80(22):11398–403. 10.1128/JVI.01177-06 16956940 PMC1642188

[135] Liu C Chen H Jia J Hong T Wang C. DCs sensitized with mPD-L1-Ig fusion protein improve the effect of heart transplantation in mice by promoting the generation of T-reg cells. Cell Immunol (2014) 290(1):169–77. 10.1016/j.cellimm.2014.04.005 24997656

[136] Blazar BR Carreno BM Panoskaltsis-Mortari A Carter L Iwai Y Yagita H Blockade of programmed death-1 engagement accelerates graft-versus-host disease lethality by an IFN-gamma-dependent mechanism. J Immunol (2003) 171(3):1272–7. 10.4049/jimmunol.171.3.1272 12874215

[137] Saha A O’Connor RS Thangavelu G Lovitch SB Dandamudi DB Wilson CB Programmed death ligand-1 expression on donor T cells drives graft-versus-host disease lethality. J Clin Invest (2016) 126:2642–60. 10.1172/JCI85796 27294527 PMC4922691

[138] Ansari MJ Salama AD Chitnis T Smith RN Yagita H Akiba H The programmed death-1 (PD-1) pathway regulates autoimmune diabetes in nonobese diabetic (NOD) mice. J Exp Med (2003) 198(1):63–9. 10.1084/jem.20022125 12847137 PMC2196083

[139] Martin-Orozco N Wang YH Yagita H Dong C. Cutting edge: programmed death (PD) ligand-1/PD-1 interaction is required for CD8+ T cell tolerance to tissue antigens. J Immunol (2006) 177(12):8291–5. 10.4049/jimmunol.177.12.8291 17142723

[140] Prokunina L Castillejo-López C Oberg F Gunnarsson I Berg L Magnusson V A regulatory polymorphism in PDCD1 is associated with susceptibility to systemic lupus erythematosus in humans. Nat Genet (2002) 32(4):666–9. 10.1038/ng1020 12402038

[141] Dong H Strome SE Salomao DR Tamura H Hirano F Flies DB Tumor-associated B7-H1 promotes T-cell apoptosis: a potential mechanism of immune evasion. Nat Med (2002) 8(8):793–800. 10.1038/nm0902-1039c 12091876

[142] Iwai Y Ishida M Tanaka Y Okazaki T Honjo T Minato N. Involvement of PD-L1 on tumor cells in the escape from host immune system and tumor immunotherapy by PD-L1 blockade. Proc Natl Acad Sci U S A (2002) 99(19):12293–7. 10.1073/pnas.192461099 12218188 PMC129438

[143] Thompson RH Gillett MD Cheville JC Lohse CM Dong H Webster WS Costimulatory B7-H1 in renal cell carcinoma patients: indicator of tumor aggressiveness and potential therapeutic target. Proc Natl Acad Sci U S A (2004) 101(49):17174–9. 10.1073/pnas.0406351101 15569934 PMC534606

[144] Ohigashi Y Sho M Yamada Y Tsurui Y Hamada K Ikeda N Clinical significance of programmed death-1 ligand-1 and programmed death-1 ligand-2 expression in human esophageal cancer. Clin Cancer Res (2005) 11(8):2947–53. 10.1158/1078-0432.CCR-04-1469 15837746

[145] Wu C Zhu Y Jiang J Zhao J Zhang XG Xu N. Immunohistochemical localization of programmed death-1 ligand-1 (PD-L1) in gastric carcinoma and its clinical significance. Acta Histochem (2006) 108(1):19–24. 10.1016/j.acthis.2006.01.003 16530813

[146] Hamanishi J Mandai M Iwasaki M Okazaki T Tanaka Y Yamaguchi K Programmed cell death 1 ligand 1 and tumor-infiltrating CD8+ T lymphocytes are prognostic factors of human ovarian cancer. Proc Natl Acad Sci U S A (2007) 104(9):3360–5. 10.1073/pnas.0611533104 17360651 PMC1805580

[147] Nakanishi J Wada Y Matsumoto K Azuma M Kikuchi K Ueda S. Overexpression of B7-H1 (PD-L1) significantly associates with tumor grade and postoperative prognosis in human urothelial cancers. Cancer Immunol Immunother (2007) 56(8):1173–82. 10.1007/s00262-006-0266-z 17186290 PMC11029839

[148] Nomi T Sho M Akahori T Hamada K Kubo A Kanehiro H Clinical significance and therapeutic potential of the programmed death-1 ligand/programmed death-1 pathway in human pancreatic cancer. Clin Cancer Res (2007) 13(7):2151–7. 10.1158/1078-0432.CCR-06-2746 17404099

[149] Hino R Kabashima K Kato Y Yagi H Nakamura M Honjo T Tumor cell expression of programmed cell death-1 ligand 1 is a prognostic factor for malignant melanoma. Cancer (2010) 116(7):1757–66. 10.1002/cncr.24899 20143437

[150] Robert C Long GV Brady B Dutriaux C Maio M Mortier L Nivolumab in previously untreated melanoma without BRAF mutation. N Engl J Med (2015) 372(4):320–30. 10.1056/NEJMoa1412082 25399552

[151] Herbst RS Soria JC Kowanetz M Fine GD Hamid O Gordon MS Predictive correlates of response to the anti-PD-L1 antibody MPDL3280A in cancer patients. Nature (2014) 515(7528):563–7. 10.1038/nature14011 25428504 PMC4836193

[152] Marzec M Zhang Q Goradia A Raghunath PN Liu X Paessler M Oncogenic kinase NPM/ALK induces through STAT3 expression of immunosuppressive protein CD274 (PD-L1, B7-H1). Proc Natl Acad Sci U S A (2008) 105(52):20852–7. 10.1073/pnas.0810958105 19088198 PMC2634900

[153] Taube JM Anders RA Young GD Xu H Sharma R McMiller TL Colocalization of inflammatory response with B7-h1 expression in human melanocytic lesions supports an adaptive resistance mechanism of immune escape. Sci Transl Med (2012) 4(127):127ra37. 10.1126/scitranslmed.3003689 22461641 PMC3568523

[154] Azuma T Yao S Zhu G Flies AS Flies SJ Chen L. B7-H1 is a ubiquitous antiapoptotic receptor on cancer cells. Blood (2008) 111(7):3635–43. 10.1182/blood-2007-11-123141 18223165 PMC2275025

[155] Ahmadzadeh M Johnson LA Heemskerk B Wunderlich JR Dudley ME White DE Tumor antigen-specific CD8 T cells infiltrating the tumor express high levels of PD-1 and are functionally impaired. Blood (2009) 114(8):1537–44. 10.1182/blood-2008-12-195792 19423728 PMC2927090

[156] Brahmer JR Drake CG Wollner I Powderly JD Picus J Sharfman WH Phase I study of single-agent anti-programmed death-1 (MDX-1106) in refractory solid tumors: safety, clinical activity, pharmacodynamics, and immunologic correlates. J Clin Oncol (2010) 28(19):3167–75. 10.1200/JCO.2009.26.7609 20516446 PMC4834717

[157] Motzer RJ Escudier B McDermott DF George S Hammers HJ Srinivas S Nivolumab versus everolimus in advanced renal-cell carcinoma. N Engl J Med (2015) 373(19):1803–13. 10.1056/NEJMoa1510665 26406148 PMC5719487

[158] Robert C Schachter J Long GV Arance A Grob JJ Mortier L Pembrolizumab versus ipilimumab in advanced melanoma. N Engl J Med (2015) 372(26):2521–32. 10.1056/NEJMoa1503093 25891173

[159] Nghiem PT Bhatia S Lipson EJ Kudchadkar RR Miller NJ Annamalai L PD-1 blockade with pembrolizumab in advanced merkel-cell carcinoma. N Engl J Med (2016) 374(26):2542–52. 10.1056/NEJMoa1603702 27093365 PMC4927341

[160] Powles T Eder JP Fine GD Braiteh FS Loriot Y Cruz C MPDL3280A (anti-PD-L1) treatment leads to clinical activity in metastatic bladder cancer. Nature (2014) 515(7528):558–62. 10.1038/nature13904 25428503

[161] Lipson EJ Sharfman WH Drake CG Wollner I Taube JM Anders RA Durable cancer regression off-treatment and effective reinduction therapy with an anti-PD-1 antibody. Clin Cancer Res (2013) 19(2):462–8. 10.1158/1078-0432.CCR-12-2625 23169436 PMC3548952

[162] Le DT Uram JN Wang H Bartlett BR Kemberling H Eyring AD PD-1 blockade in tumors with mismatch-repair deficiency. N Engl J Med (2015) 372(26):2509–20. 10.1056/NEJMoa1500596 26028255 PMC4481136

[163] Armand P Nagler A Weller EA Devine SM Avigan DE Chen YB Disabling immune tolerance by programmed death-1 blockade with pidilizumab after autologous hematopoietic stem-cell transplantation for diffuse large B-cell lymphoma: results of an international phase II trial. J Clin Oncol (2013) 31(33):4199–206. 10.1200/JCO.2012.48.3685 24127452 PMC4878008

[164] Westin JR Chu F Zhang M Fayad LE Kwak LW Fowler N Safety and activity of PD1 blockade by pidilizumab in combination with rituximab in patients with relapsed follicular lymphoma: a single group, open-label, phase 2 trial. Lancet Oncol (2014) 15(1):69–77. 10.1016/S1470-2045(13)70551-5 24332512 PMC3922714

[165] Hamid O Robert C Daud A Hodi FS Hwu WJ Kefford R Safety and tumor responses with lambrolizumab (anti-PD-1) in melanoma. N Engl J Med (2013) 369(2):134–44. 10.1056/NEJMoa1305133 23724846 PMC4126516

[166] Ribas A Hodi FS Kefford R Hamid O Daud A Wolchok JD Efficacy and safety of the anti-PD-1 monoclonal antibody MK-3475 in 411 patients (pts) with melanoma (MEL). J Clin Oncol (2014) 32(5s):suppl; abstr LBA9000^.

[167] Weber JS Kudchadkar RR Yu B Gallenstein D Horak CE Inzunza HD Safety, efficacy, and biomarkers of nivolumab with vaccine in ipilimumab-refractory or -naive melanoma. J Clin Oncol (2013) 31(34):4311–8. 10.1200/JCO.2013.51.4802 24145345 PMC3837092

[168] Topalian SL Sznol M McDermott DF Kluger HM Carvajal RD Sharfman WH Survival, durable tumor remission, and long-term safety in patients with advanced melanoma receiving nivolumab. J Clin Oncol (2014) 32(10):1020–30. 10.1200/JCO.2013.53.0105 24590637 PMC4811023

[169] Robert C Ribas A Wolchok JD Hodi FS Hamid O Kefford R Anti-programmed-death-receptor-1 treatment with pembrolizumab in ipilimumab-refractory advanced melanoma: a randomised dose-comparison cohort of a phase 1 trial. Lancet (2014) 384(9948):1109–17. 10.1016/S0140-6736(14)60958-2 25034862

[170] Larkin J Chiarion-Sileni V Gonzalez R Grob JJ Cowey CL Lao CD Combined nivolumab and ipilimumab or monotherapy in untreated melanoma. N Engl J Med (2015) 373(1):23–34. 10.1056/NEJMoa1504030 26027431 PMC5698905

[171] Weber JS D’Angelo SP Minor D Hodi FS Gutzmer R Neyns B Nivolumab versus chemotherapy in patients with advanced melanoma who progressed after anti-CTLA-4 treatment (CheckMate 037): a randomised, controlled, open-label, phase 3 trial. Lancet Oncol (2015) 16(4):375–84. 10.1016/S1470-2045(15)70076-8 25795410

[172] Brahmer J Reckamp KL Baas P Crinò L Eberhardt WE Poddubskaya E Nivolumab versus docetaxel in advanced squamous-cell non-small-cell lung cancer. N Engl J Med (2015) 373(2):123–35. 10.1056/NEJMoa1504627 26028407 PMC4681400

[173] Gettinger SN Horn L Gandhi L Spigel DR Antonia SJ Rizvi NA Overall survival and long-term safety of nivolumab (anti-programmed death 1 antibody, BMS-936558, ONO-4538) in patients with previously treated advanced non-small-cell lung cancer. J Clin Oncol (2015) 33(18):2004–12. 10.1200/JCO.2014.58.3708 25897158 PMC4672027

[174] Rizvi NA Mazières J Planchard D Stinchcombe TE Dy GK Antonia SJ Activity and safety of nivolumab, an anti-PD-1 immune checkpoint inhibitor, for patients with advanced, refractory squamous non-small-cell lung cancer (CheckMate 063): a phase 2, single-arm trial. Lancet Oncol (2015) 16(3):257–65. 10.1016/S1470-2045(15)70054-9 25704439 PMC5726228

[175] Hodi FS O’Day SJ McDermott DF Weber RW Sosman JA Haanen JB Improved survival with ipilimumab in patients with metastatic melanoma. N Engl J Med (2010) 363(8):711–23. 10.1056/NEJMoa1003466 20525992 PMC3549297

[176] Ribas A Puzanov I Dummer R Schadendorf D Hamid O Robert C Pembrolizumab versus investigator-choice chemotherapy for ipilimumab-refractory melanoma (KEYNOTE-002): a randomised, controlled, phase 2 trial. Lancet Oncol (2015) 16(8):908–18. 10.1016/S1470-2045(15)00083-2 26115796 PMC9004487

[177] Borghaei H Paz-Ares L Horn L Spigel DR Steins M Ready NE Nivolumab versus docetaxel in advanced nonsquamous non-small-cell lung cancer. N Engl J Med (2015) 373(17):1627–39. 10.1056/NEJMoa1507643 26412456 PMC5705936

[178] Ding H Wu X Gao W. PD-L1 is expressed by human renal tubular epithelial cells and suppresses T cell cytokine synthesis. Clin Immunol (2005) 115(2):184–91. 10.1016/j.clim.2005.01.005 15885642

[179] Motzer RJ Rini BI McDermott DF Redman BG Kuzel TM Harrison MR Nivolumab for metastatic renal cell carcinoma: results of a randomized phase II trial. J Clin Oncol (2015) 33(13):1430–7. 10.1200/JCO.2014.59.0703 25452452 PMC4806782

[180] Amin A Plimack ER Infante JR Ernstoff MS Rini BI McDermott DF Nivolumab (anti-PD-1; BMS-936558, ONO-4538) in combination with sunitinib or pazopanib in patients (pts) with metastatic renal cell carcinoma (mRCC). J Clin Oncol (2014) 32(Suppl 5s):abstr 5010.

[181] Armand P Shipp MA Ribrag V Michot JM Zinzani PL Kuruvilla J Programmed death-1 blockade with pembrolizumab in patients with classical Hodgkin lymphoma after brentuximab vedotin failure. J Clin Oncol (2016) 34(31):3733–39. 10.1200/JCO.2016.67.3467 27354476 PMC5791838

[182] Moskowitz CH Ribrag V Michot JM Martinelli G Zinzani PL Gutierrez M PD-1 blockade with the monoclonal antibody pembrolizumab (MK-3475) in patients with classical Hodgkin lymphoma after brentuximab vedotin failure: preliminary results from a phase 1b study (KEYNOTE-013). Blood (2014) 124:290.

[183] Green MR Monti S Rodig SJ Juszczynski P Currie T O’Donnell E Integrative analysis reveals selective 9p24.1 amplification, increased PD-1 ligand expression, and further induction via JAK2 in nodular sclerosing Hodgkin lymphoma and primary mediastinal large B-cell lymphoma. Blood (2010) 116(17):3268–77. 10.1182/blood-2010-05-282780 20628145 PMC2995356

[184] Philips GK Atkins M. Therapeutic uses of anti-PD-1 and anti-PD-L1 antibodies. Int Immunol (2015) 27(1):39–46. 10.1093/intimm/dxu095 25323844

[185] Spira AI Park K Mazières J Vansteenkiste JF Rittmeyer A Ballinger M Efficacy, safety and predictive biomarker results from a randomized phase II study comparing MPDL3280A vs docetaxel in 2L/3L NSCLC (POPLAR). J Clin Oncol (2015) 33:abstr 8010.

[186] Rizvi NA Brahmer JR Ou SHI Segal NH Khleif S Hwu WJ Safety and clinical activity of MEDI4736, an anti-programmed cell death-ligand 1 (PD-L1) antibody, in patients with non-small cell lung cancer (NSCLC). J Clin Oncol (2015) 33:abstr 8032

[187] Wolchok JD Kluger H Callahan MK Postow MA Rizvi NA Lesokhin AM Nivolumab plus ipilimumab in advanced melanoma. N Engl J Med (2013) 369(2):122–33. 10.1056/NEJMoa1302369 23724867 PMC5698004

[188] Postow MA Chesney J Pavlick AC Robert C Grossmann K McDermott D Nivolumab and ipilimumab versus ipilimumab in untreated melanoma. N Engl J Med (2015) 372(21):2006–17. 10.1056/NEJMoa1414428 25891304 PMC5744258

[189] Hodi FS Chesney J Pavlick AC Robert C Grossmann KF McDermott DF Combined nivolumab and ipilimumab versus ipilimumab alone in patients with advanced melanoma: 2-year overall survival outcomes in a multicentre, randomised, controlled, phase 2 trial. Lancet Oncol (2016) 17:1558–68. 10.1016/S1470-2045(16)30366-7 27622997 PMC5630525

[190] Hammers HJ Plimack ER Infante JR Ernstoff MS Rini BI McDermott DF Phase I study of nivolumab in combination with ipilimumab in metastatic renal cell carcinoma (mRCC). J Clin Oncol (2014) 32(Suppl 5s):abstr 4504. 10.1200/JCO.2016.72.1985 PMC7587408 28678668

[191] Antonia SJ Goldberg SB Balmanoukian AS Sanborn RE Steele K Narwal R Phase Ib study of MEDI4736, a programmed cell death ligand-1 (PD-L1) antibody, in combination with tremelimumab, a cytotoxic T-lymphocyte-associated protein-4 (CTLA-4) antibody, in patients (pts) with advanced NSCLC. J Clin Oncol (2015) 33:abstr 3014.

[192] Riley JL Combination checkpoint blockade – taking melanoma immunotherapy to the next level. N Engl J Med (2013) 369(2):187–9. 10.1056/NEJMe1305484 23724866

[193] Tumeh PC Harview CL Yearley JH Shintaku IP Taylor EJ Robert L PD-1 blockade induces responses by inhibiting adaptive immune resistance. Nature (2014) 515(7528):568–71. 10.1038/nature13954 25428505 PMC4246418

[194] Cho DC Sosman JA Sznol M Gordon MS Hollebecque A Hamid O Clinical activity, safety, and biomarkers of MPDL3280A, an engineered PD-L1 antibody in patients with metastatic renal cell carcinoma (mRCC). J Clin Oncol (2013) 31:abstr 4505.

[195] Fridman WH Pagès F Sautès-Fridman C Galon J. The immune contexture in human tumours: impact on clinical outcome. Nat Rev Cancer (2012) 12(4):298–306. 10.1038/nrc3245 22419253

[196] Galon J Angell HK Bedognetti D Marincola FM. The continuum of cancer immunosurveillance: prognostic, predictive, and mechanistic signatures. Immunity (2013) 39(1):11–26. 10.1016/j.immuni.2013.07.008 23890060

[197] Ogino S Galon J Fuchs CS Dranoff G Cancer immunology – analysis of host and tumor factors for personalized medicine. Nat Rev Clin Oncol (2011) 8(12):711–9. 10.1038/nrclinonc.2011.122 21826083 PMC3227751

[198] Kohrt HE Tumeh PC Benson D Bhardwaj N Brody J Formenti S Immunodynamics: a cancer immunotherapy trials network review of immune monitoring in immuno-oncology clinical trials. J Immunother Cancer (2016) 4:15. 10.1186/s40425-016-0118-0 26981245 PMC4791805

